# Dynamic Multi-Image Weighting for Automated Detection and Diagnosis of Abnormal Urinary Tract on Voiding Cystourethrography with a Deep Learning System: A Retrospective, Large-Scale, Multicenter Study

**DOI:** 10.34133/research.0771

**Published:** 2025-07-22

**Authors:** Min Wu, Zhanchi Li, YiDong Liu, Zelong Tan, Wenjuan Tang, Xiaoqi Xuan, Hui Feng, Weihua Lao, Ning Ding, BoJun Wang, Zheyuan Wang, Likai Zhuang

**Affiliations:** ^1^ Department of Urology, Shanghai Children’s Hospital, School of Medicine, Shanghai Jiao Tong University, Shanghai 200062, China.; ^2^ Department of Urology, Shanghai Punan Hospital of Pudong New District, Punan Branch of Renji Hospital, Shanghai 200000, China.; ^3^ Department of Urology, Renji Hospital, School of Medicine, Shanghai Jiao Tong University, Shanghai, China.; ^4^ Department of Electronic Engineering, Tsinghua University, Beijing, China.; ^5^ Department of Radiology, Shanghai Children’s Hospital, School of Medicine, Shanghai Jiao Tong University, Shanghai 200062, China.; ^6^ Department of Urology, Wuxi Children’s Hospital, Wuxi, China.; ^7^ Department of Urology, The Sixth Affiliated Hospital of Harbin Medical University, Harbin, China.; ^8^ Department of Urology, Guangdong Women and Children Hospital, Guangzhou, China.; ^9^ Department of Urology, Jiangxi Provincial Children’s Hospital, Nanchang, China.; ^10^ Department of Radiology, Children′s Hospital of Fudan University, Shanghai 201102, China.; ^11^ Department of Computer Science and Engineering, Shanghai Jiao Tong University, Shanghai 200240, China.; ^12^ Department of Urology, Children′s Hospital of Fudan University, Shanghai 201102, China.

## Abstract

We aimed to develop a voiding cystourethrography (VCUG) diagnostic artificial intelligence model (VCUG-DAM), which relies on a novel architecture to automatically segment and diagnose the bladder, urethra, and ureters using a single VCUG image, while dynamically assessing the relative importance of each image. A total of 7,899 VCUG images from 1,660 patients across 15 Chinese hospitals were collected between 2021 and 2023. In stage 1, we assessed the performances of the VCUG-DAM model. The patient-level area under the curve (AUC) of VCUG-DAM was 0.8772, 0.7752, 0.9443, and 0.9342 for bladder, urethral, left, and right vesicoureteral reflux (VUR), respectively. In stage 2, we explored whether the VCUG-DAM model could improve the diagnostic ability of clinicians. VCUG-DAM improved the clinician’s diagnostic performance, with mean AUCs increasing from 0.8185 to 0.9456 for the bladder, 0.6507 to 0.7943 for the urethra, 0.6288 to 0.9641 for the left VUR, and 0.7305 to 0.9506 for the right VUR (all *P* < 0.0001). In stage 3, the consistency of the VCUG-DAM for VUR grading was validated. VCUG-DAM improved inter-clinician agreement for VUR grading. The fully automated VCUG-DAM demonstrated high accuracy, reliability, and robustness in multitask diagnoses of urinary tract abnormalities across multiple VCUG images, while improving the diagnostic ability of clinicians as an auxiliary tool.

## Introduction

Voiding cystourethrography (VCUG) is a widely used pediatric fluoroscopy technique that provides detailed anatomical and functional information about the urethra, bladder, and ureters during bladder filling and emptying [1–3]. Contrast-enhanced voiding ultrasonography (ceVUS) has nowadays almost completely replaced VCUG, especially in the diagnosis of vesicoureteral reflux (VUR), but together with ceVUS, VCUG remains the gold standard test to diagnose VUR. It is one of the most performed fluoroscopic examinations in pediatric radiology departments [2]. During the filling phase, VCUG can help in assessing the bladder’s shape, size, and filling defects, and in identifying VUR. In the voiding phase, VCUG allows simultaneous evaluation of reflux and potential anatomical abnormalities or dysfunctions such as bladder diverticula, trabeculations [4], ureterocele [5], posterior urethral valve [6], anterior urethral valve [7], and urethral stenosis [8,9]. However, diagnostic accuracy can be influenced by variations in techniques and interobserver variability.

Recent advancements in deep learning (DL) have demonstrated higher efficiency and reproducibility than those of traditional radiomics methods, which rely on handcrafted features for medical imaging analysis [10–12]. Eroglu et al. [13] were the first to propose a convolutional neural network (CNN) model for grading VUR on VCUG images. In our preliminary research [14], we developed a voting ensemble learning method capable of predicting unilateral or bilateral VUR grades with high objectivity and reliability. However, existing CNN models focus solely on VUR grading and fail to address bladder and urethral abnormalities. Additionally, current DL approaches for VUR grading require manual selection of VCUG images, further limiting their clinical applicability.

To address these limitations, we aimed to develop a VCUG diagnostic artificial intelligence (AI) model (VCUG-DAM), which relies on a novel architecture to automatically segment and diagnose the bladder, urethra, and ureters using a single VCUG image. By aggregating information from multiple images, VCUG-DAM calculates the relative importance of each image to generate a comprehensive diagnosis for individual patients. Furthermore, we evaluated the diagnostic performance of VCUG-DAM against that of 12 clinicians to determine whether it could enhance accuracy and agreement.

## Results

A total of 1,270 VCUG examinations (5,644 images) were collected from the Children’s Hospital of Fudan University. The participants had a mean age of 42.12 ± 40.78 months, of whom 780 (61.42%) were males and 490 (38.58%) were females. External validation dataset 1 featured 256 VCUG examinations (1,694 images) from 10 hospitals, and the participants had a mean age of 40.97±42.18 months, of whom 239 (62.73%) were males and 142 (37.27%) were females. External validation dataset 2 featured 134 VCUG examinations (490 images) from 4 hospitals, and the participants had a mean age of 41.34±29.91 months, of whom 82 (61.19%) were males and 52 (38.81%) were females. Table 1 provides a detailed overview of the demographic characteristics of the study participants.

**Table 1.** Baseline characteristics of cohorts. Data are presented as *n* (%).

Participant characteristics	Training dataset		Internal validation dataset		External validation dataset 1		External validation dataset 2	
Patients	889		381		256		134	
Voiding cystourethrography	889		381		256		134	
Age (month, mean ± SD)	42.64 ± 40.14		40.97 ± 42.18		45.20 ± 35.89		41.34 ± 29.91	
Sex								
Male	541 (60.85%)		239 (62.73%)		134 (52.34%)		82 (61.19%)	
Female	348 (39.15%)		142 (37.27%)		122 (47.66%)		52 (38.81%)	
Number of VCUG images	3,950		1,694		1,765		490	
Labeled VCUG image categories								
	**Left**	**Right**	**Left**	**Right**	**Left**	**Right**	**Left**	**Right**
VUR-Grade 0	3,016	3,464	1,270	1,484	179	193	54	61
VUR-Grade 1	135	60	56	29	8	5	3	1
VUR-Grade 2	63	82	31	31	6	9	6	6
VUR-Grade 3	352	173	176	76	41	28	41	39
VUR-Grade 4	274	82	110	38	17	15	18	20
VUR-Grade 5	110	89	51	36	5	6	12	7
Bladder—abnormal	500		204		24		–	
Bladder—normal	3,450		1,490		232		–	
Urethra—abnormal	173		73		13		–	
Urethra—normal	3,777		1,621		243		–	

SD, standard deviation

### Performance of VCUG-DAM at image-level and patient-level diagnoses

**Fig. 1.**
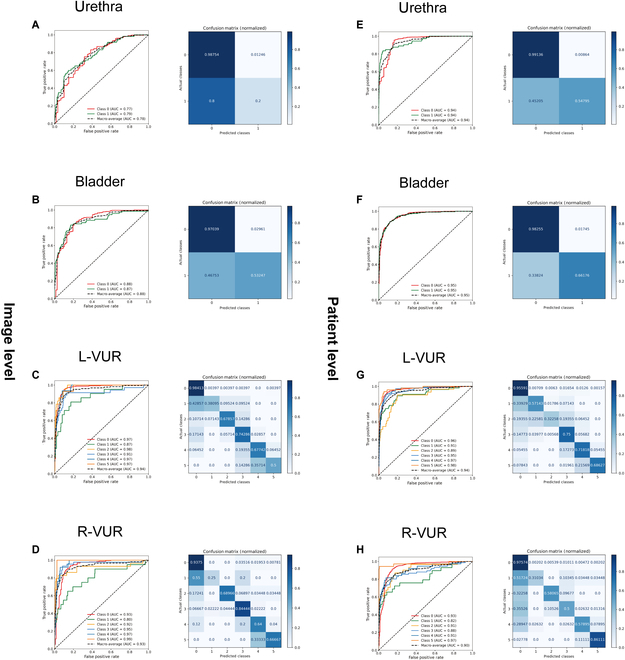
Confusion matrices and ROC curvature of the VCUG-DAM performance at the image level (A to D) and patient level (E to H) in the internal dataset. L-VUR, the grading of the left VUR; R-VUR, the grading of the right VUR.

In stage 1, we used the internal validation dataset to evaluate the performance of VCUG-DAM at the image and patient levels (Fig. 1). For the image level, the overall performance of the bladder classification showed an accuracy of 0.994 (95% confidence interval: [CI]: 0.932 to 0.9545) and an area under the curve (AUC) of 0.9507 (95% CI: 0.9340 to 0.9650). The overall performance of the urethra classification showed an accuracy of 0.9723 (95% CI: 0.9640 to 0.9799) and an AUC of 0.9408 (95% CI: 0.9112 to 0.9674). The overall performance of the left (L)-VUR and right (R)-VUR classifications showed an accuracy of 0.8868 (95% CI: 0.8719 to 0.9020) and 0.9246 (95% CI: 0.9115 to 0.9368), respectively, with corresponding AUCs of 0.9568 (95% CI: 0.9463 to 0.9662) and 0.9249 (95% CI: 0.9006 to 0.9465).

At the patient level, the overall performance of the bladder classification showed an accuracy of 0.8740 (95% CI: 0.8399 to 0.9081) and an AUC of 0.8772 (95% CI: 0.8279 to 0.9265). The overall performance of the urethra classification showed an accuracy of 0.8661 (95% CI: 0.8320 to 0.9003) and an AUC of 0.7752 (95% CI: 0.7094 to 0.8410). The overall performance of the L-VUR and R-VUR classifications showed an accuracy of 0.8570 (95% CI: 0.8215 to 0.8924) and 0.8517 (95% CI: 0.8163 to 0.8871), respectively, with corresponding AUCs of 0.9443 (95% CI: 0.9242 to 0.9644) and 0.9342 (95% CI: 0.9065 to 0.9618). Detailed evaluation metrics, including accuracy, sensitivity, specificity, and F1 scores for each condition, are provided in Appendix Tables S3 and S4.

### Clinician’s performance with and without VCUG-DAM assistance at patient-level diagnosis

**Fig. 2.**
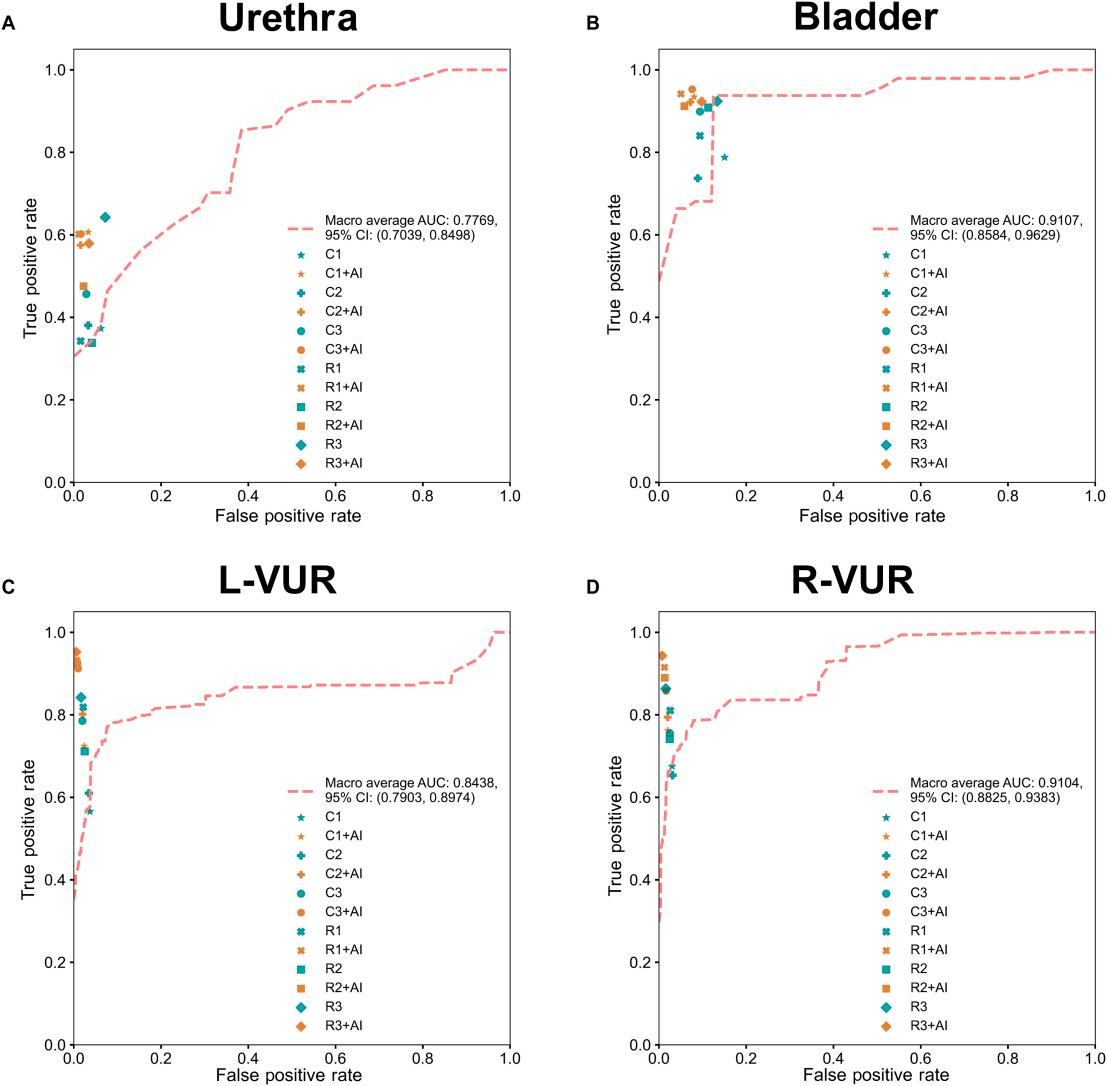
Performance of the VCUG-DAM model and clinicians with and without model assistance in classification. (A) Urethra. (B) Bladder. (C) Left-VUR. (D) Right-VUR. AI, VCUG-DAM; C1, mean junior clinicians; C2, mean attending clinicians; C3, mean senior clinicians; R1, mean junior radiologist; R2, mean attending radiologist; R3, mean senior radiologist. There were 2 senior clinicians, 2 attending clinicians, and 2 junior clinicians or radiologists in each group. The blue dots denote the performance of the clinicians without assistance from the VCUG-DAM model. The gray dots denote the performance of the clinicians with assistance from the VCUG-DAM model.

In stage 2, we assessed clinician performance with and without VCUG-DAM assistance for patient-level diagnosis using the external validation dataset (Fig. 2, Table 2, and Tables S5 to S8). The VCUG-DAM attained a mean AUC of 0.8438 (95% CI: 0.7903 to 0.8974) and 0.9104 (95% CI: 0.8825 to 0.9383) in the grading of L-VUR and R-VUR, respectively. For distinguishing bladder and urethral, the mean AUC of VCUG-DAM was 0.9107 (95% CI: 0.8584 to 0.9629) and 0.7769 (95% CI: 0.7039 to 0.8498), respectively. VCUG-DAM improved the diagnostic ability of clinicians before and after assistance, with AUC comparisons of 0.8185 (95% CI: 0.7756 to 0.8615) versus 0.9456 (95% CI: 0.9261 to 0.9651) for bladder (*P* < 0.0001); 0.6507 (95% CI: 0.5837 to 0.7176) versus 0.7943 (95% CI: 0.7293 to 0.8592) for urethra (*P* < 0.0001); 0.6288 (95% CI: 0.5776 to 0.6801) versus 0.9641 (95% CI: 0.9406 to 0.9875) for L-VUR grading (*P* < 0.0001); and 0.7305 (95% CI: 0.6793 to 0.7817) versus 0.9506 (95% CI: 0.9221 to 0.9791) for R-VUR grading (*P* < 0.0001), respectively.

**Table 2.** The performance of VCUG-DAM, clinicians alone, and VCUG-DAM-assisted clinicians in external validation dataset 1

	Accuracy (95% CI)				AUC (95% CI)				Sensitivity (95% CI)				Specificity (95% CI)			
	Bladder	Urethral	L-VUR	R-VUR	Bladder	Urethral	L-VUR	R-VUR	Bladder	Urethral	L-VUR	R-VUR	Bladder	Urethral	L-VUR	R-VUR
VCUG-DAM model	0.9883 (0.9805, 0.9961)	0.9707 (0.9609, 0.9805)	0.9098 (0.8900, 0.9297)	0.8965 (0.8750, 0.9180)	0.9107 (0.8584, 0.9629)	0.7769 (0.7039, 0.8498)	0.8438 (0.7903, 0.8974)	0.9104 (0.8825, 0.9383)	0.8731 (0.7917, 0.9545)	0.4295 (0.2857, 0.5733)	0.7259 (0.6565, 0.7953)	0.7539 (0.6878, 0.8200)	1.0000 (1.0000, 1.0000)	1.0000 (1.0000, 1.0000)	0.9673 (0.9603, 0.9742)	0.9699 (0.9624, 0.9775)
Clinicians without VCUG-DAM assistance																
Mean senior clinicians	0.843 (0.8223, 0.8636)	0.9091 (0.8903, 0.9278)	0.8311 (0.8103, 0.8519)	0.8750 (0.8552, 0.8948)	0.8185 (0.7756, 0.8615)	0.6572 (0.5903, 0.7239)	0.6288 (0.5776, 0.6801)	0.7499 (0.7037, 0.7961)	0.7878 (0.7051, 0.8704)	0.3730 (0.2374, 0.5086)	0.5657 (0.5044, 0.6271)	0.6754 (0.6161, 0.7347)	0.8495 (0.8284, 0.8705)	0.9381 (0.9231, 0.9531)	0.9629 (0.9574, 0.9685)	0.9699 (0.9643, 0.9755)
Mean attending clinicians	0.8955 (0.8750, 0.9160)	0.9344 (0.9177, 0.9511)	0.8504 (0.8295, 0.8714)	0.8623 (0.8454, 0.8792)	0.8263 (0.7809, 0.8716)	0.6746 (0.6118, 0.7375)	0.6741 (0.6228, 0.7254)	0.7305 (0.6793, 0.7817)	0.7369 (0.6472, 0.8267)	0.3805 (0.2609, 0.5000)	0.6099 (0.5508, 0.6691)	0.6533 (0.5988, 0.7079)	0.9107 (0.8922, 0.9294)	0.9668 (0.9545, 0.9792)	0.9656 (0.9598, 0.9715)	0.9688 (0.9647, 0.9728)
Mean junior clinicians	0.9054 (0.8867, 0.9242)	0.9462 (0.9314, 0.9609)	0.9120 (0.8942, 0.9296)	0.8965 (0.8805, 0.9124)	0.9040 (0.8744, 0.9336)	0.7152 (0.6468, 0.7836)	0.8246 (0.7785, 0.8707)	0.8102 (0.7618, 0.8586)	0.8988 (0.8403, 0.9572)	0.4564 (0.3185, 0.5943)	0.7855 (0.7311, 0.8399)	0.7546 (0.6937, 0.8155)	0.9061 (0.8858, 0.9264)	0.9712 (0.9609, 0.9816)	0.9803 (0.9761, 0.9845)	0.9751 (0.9698, 0.9804)
Mean senior radiologist	0.9020 (0.8861, 0.9180)	0.9510 (0.9391, 0.9628)	0.8838 (0.8669, 0.9007)	0.8660 (0.8454, 0.8867)	0.8740 (0.8395, 0.9083)	0.6647 (0.6004, 0.7291)	0.8376 (0.7935, 0.8817)	0.8507 (0.8085, 0.8928)	0.8402 (0.7775, 0.9028)	0.3425 (0.2167, 0.4682)	0.8186 (0.7699, 0.8674)	0.8104 (0.7575, 0.8631)	0.9061 (0.8896, 0.9226)	0.9844 (0.9768, 0.9918)	0.9785 (0.9749, 0.9820)	0.9742 (0.9700, 0.9785)
Mean attending radiologist	0.8880 (0.8672, 0.9088)	0.9287 (0.9141, 0.9434)	0.8830 (0.8633, 0.9026)	0.8770 (0.8591, 0.8948)	0.9009 (0.8690, 0.9328)	0.6507 (0.5837, 0.7176)	0.7540 (0.6949, 0.8129)	0.8015 (0.7543, 0.8487)	0.9084 (0.8543, 0.9625)	0.3380 (0.2080, 0.4679)	0.7115 (0.6478, 0.7752)	0.7412 (0.6826, 0.7997)	0.8868 (0.8661, 0.9074)	0.9587 (0.9466, 0.9710)	0.9748 (0.9699, 0.9798)	0.9753 (0.9711, 0.9795)
Mean junior radiologist	0.8709 (0.8493, 0.8926)	0.9120 (0.8942, 0.9297)	0.9219 (0.9062, 0.9375)	0.9220 (0.9062, 0.9378)	0.8943 (0.8660, 0.9227)	0.7830 (0.7211, 0.8448)	0.8758 (0.8462, 0.9053)	0.8952 (0.8620, 0.9285)	0.9238 (0.8724, 0.9752)	0.6425 (0.5167, 0.7684)	0.8422 (0.7984, 0.8861)	0.8633 (0.8153, 0.9113)	0.8664 (0.8424, 0.8905)	0.9280 (0.9118, 0.9443)	0.9834 (0.9792, 0.9875)	0.9844 (0.9800, 0.9889)
Clinicians with VCUG-DAM assistance																
Mean senior clinicians	0.9209 (0.9043, 0.9375)	0.9476 (0.9355, 0.9597)	0.9052 (0.8881, 0.9222)	0.9162 (0.8984, 0.9339)	0.9274 (0.9006, 0.9543)	0.7833 (0.7113, 0.8552)	0.7711 (0.7230, 0.8192)	0.8169 (0.7752, 0.8585)	0.9345 (0.8863, 0.9829)	0.6067 (0.4618, 0.7515)	0.7242 (0.6666, 0.7816)	0.7624 (0.7073, 0.8175)	0.9194 (0.9040, 0.9350)	0.9668 (0.9563, 0.9773)	0.9762 (0.9715, 0.9809)	0.9797 (0.9748, 0.9846)
Mean attending clinicians	0.9287 (0.9122, 0.9453)	0.9647 (0.9547, 0.9747)	0.9288 (0.9141, 0.9437)	0.9133 (0.8964, 0.9300)	0.9227 (0.8908, 0.9547)	0.7775 (0.7048, 0.8503)	0.8469 (0.8042, 0.8896)	0.8453 (0.8034, 0.8872)	0.9219 (0.8598, 0.9839)	0.5748 (0.4392, 0.7104)	0.8014 (0.7480, 0.8546)	0.7941 (0.7397, 0.8485)	0.9278 (0.9121, 0.9435)	0.9844 (0.9770, 0.9919)	0.9799 (0.9750, 0.9848)	0.9798 (0.9753, 0.9842)
Mean attending clinicians	0.9267 (0.9121, 0.9414)	0.9647 (0.9547, 0.9747)	0.9629 (0.9511, 0.9746)	0.9287 (0.9121, 0.9453)	0.9381 (0.9209, 0.9551)	0.7926 (0.7201, 0.8651)	0.9342 (0.9014, 0.9668)	0.8861 (0.8434, 0.9288)	0.9527 (0.9247, 0.9808)	0.6020 (0.4562, 0.7478)	0.9122 (0.8720, 0.9523)	0.8577 (0.8068, 0.9085)	0.9238 (0.9091, 0.9385)	0.9836 (0.9776, 0.9897)	0.9901 (0.9869, 0.9932)	0.9836 (0.9792, 0.9880)
Mean senior radiologist	0.9492 (0.9355, 0.9628)	0.9688 (0.9570, 0.9805)	0.9658 (0.9550, 0.9766)	0.9287 (0.9121, 0.9453)	0.9456 (0.9261, 0.9651)	0.7943 (0.7293, 0.8592)	0.9402 (0.9052, 0.9751)	0.9287 (0.8963, 0.9612)	0.9417 (0.9060, 0.9774)	0.6018 (0.4765, 0.7272)	0.9322 (0.8926, 0.9717)	0.9152 (0.8778, 0.9528)	0.9496 (0.9352, 0.9640)	0.9885 (0.9811, 0.9959)	0.9931 (0.9909, 0.9953)	0.9874 (0.9845, 0.9905)
Mean attending radiologist	0.9403 (0.9255, 0.9550)	0.9520 (0.9391, 0.9648)	0.9727 (0.9628, 0.9825)	0.9365 (0.9219, 0.9511)	0.9297 (0.9011, 0.9583)	0.7264 (0.6547, 0.7980)	0.9408 (0.9120, 0.9696)	0.9169 (0.8797, 0.9543)	0.9119 (0.8588, 0.9651)	0.4755 (0.3319, 0.6190)	0.9230 (0.8879, 0.9582)	0.8898 (0.8401, 0.9394)	0.9416 (0.9267, 0.9566)	0.9774 (0.9692, 0.9857)	0.9917 (0.9885, 0.9949)	0.9867 (0.9830, 0.9904)
Mean junior radiologist	0.9033 (0.8848, 0.9219)	0.9445 (0.9316, 0.9573)	0.9795 (0.9708, 0.9883)	0.9580 (0.9450, 0.9710)	0.9131 (0.8821, 0.9441)	0.7691 (0.7040, 0.8341)	0.9641 (0.9406, 0.9875)	0.9506 (0.9221, 0.9791)	0.9227 (0.8672, 0.9784)	0.5792 (0.4584, 0.7000)	0.9522 (0.9237, 0.9807)	0.9431 (0.9110, 0.9753)	0.9019 (0.8831, 0.9206)	0.9651 (0.9533, 0.9770)	0.9946 (0.9922, 0.9969)	0.9926 (0.9903, 0.9949)

For bladder, the most marked AUC improvement was in senior clinicians, with an increase of 13.30% (from 0.8185 [95% CI: 0.7756 to 0.8615] to 0.9274 [95% CI: 0.9006 to 0.9543], *P* < 0.01). For the urethra, the AUCs of the senior radiologists improved by an absolute margin of 19.50% (0.6647 [95% CI: 0.6004 to 0.7291] vs. 0.7943 [95% CI: 0.7293 to 0.8592], *P* < 0.0001). For the L-VUR, the AUCs of junior clinicians improved by an absolute margin of 25.63% (0.6741 [95% CI: 0.6228 to 0.7254] vs. 0.8469 [95% CI: 0.8042 to 0.8896], *P* < 0.0001). For R-VUR, the AUCs of junior clinicians improved by an absolute margin of 15.72% (0.7305 [95% CI: 0.6793 to 0.7817] vs. 0.8453 [95% CI: 0.8034 to 0.8872], *P* < 0.0001).

### Clinician’s performance in VUR grading using VCUG-DAM assistance

**Fig. 3.**
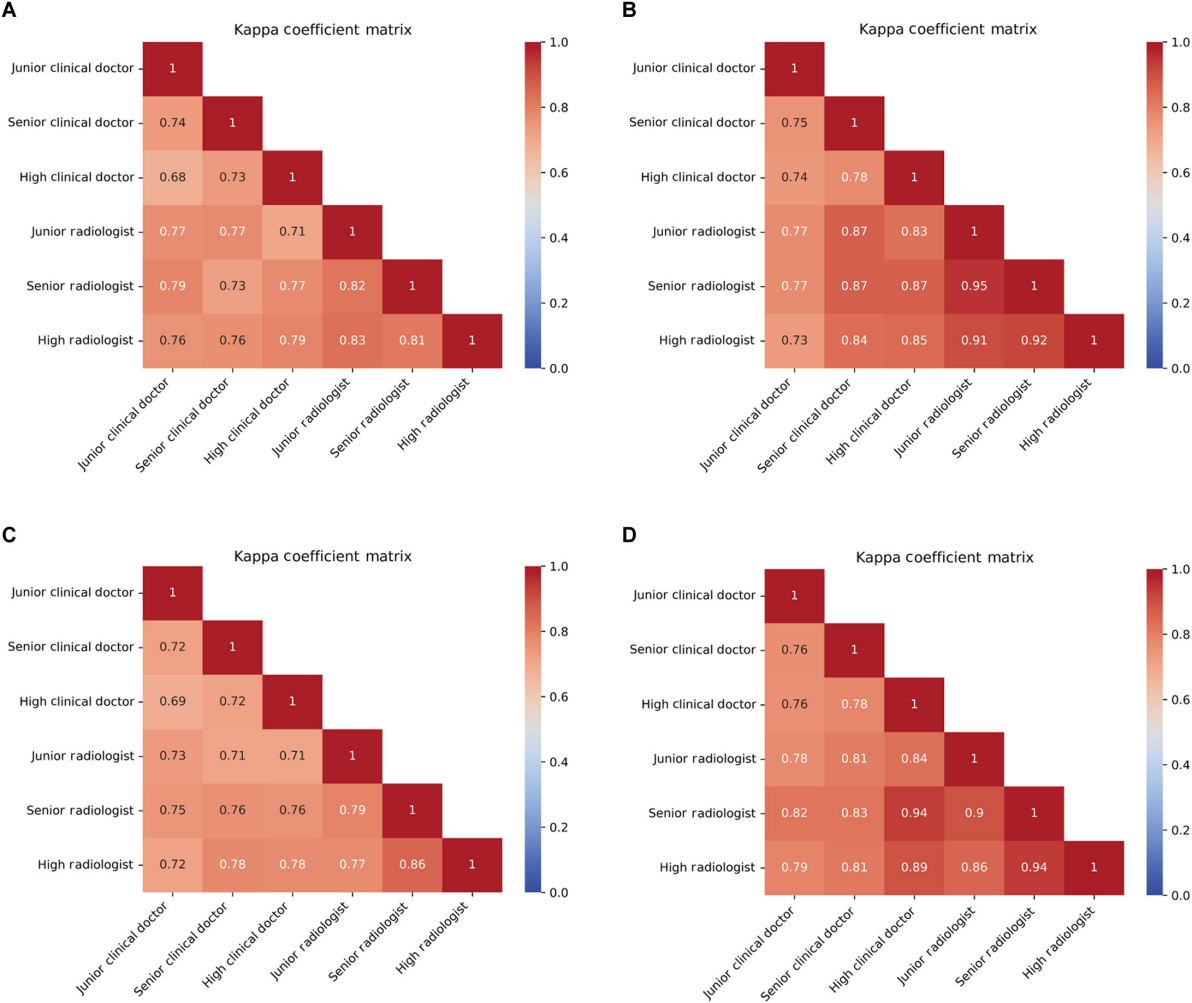
The performance of VUR grading the clinicians with and without the VCUG-DAM assist on the external validation dataset 2. Left-VUR without model assistance (A) and with model assistance (B). Right-VUR without model assistance (C) and with model assistance (D).

We compared clinician concordance in VUR grading with and without VCUG-DAM assistance using external validation dataset 2 (Fig. 3 and Tables S9 and S10). In general, clinicians using the VCUG-DAM model for L- and R-VUR grading had higher kappa, (0.73 vs. 0.87) and (0.76 vs. 0.94), respectively, than clinicians’ stand-alone diagnoses. Among these subgroups, with VCUG-DAM assistance for L-VUR grading, individual improvements ranged between 0.01 and 0.14. Senior clinical doctors achieved the highest agreement improvement (0.14), followed by inter-junior radiologists (0.13). For R-VUR grading, individual improvement ranged from 0.01 to 0.18. High clinical doctors achieved the highest agreement improvement (0.18), followed by high inter-clinical radiologists (0.11).

### Model interpretability and visualization

**Fig. 4.**
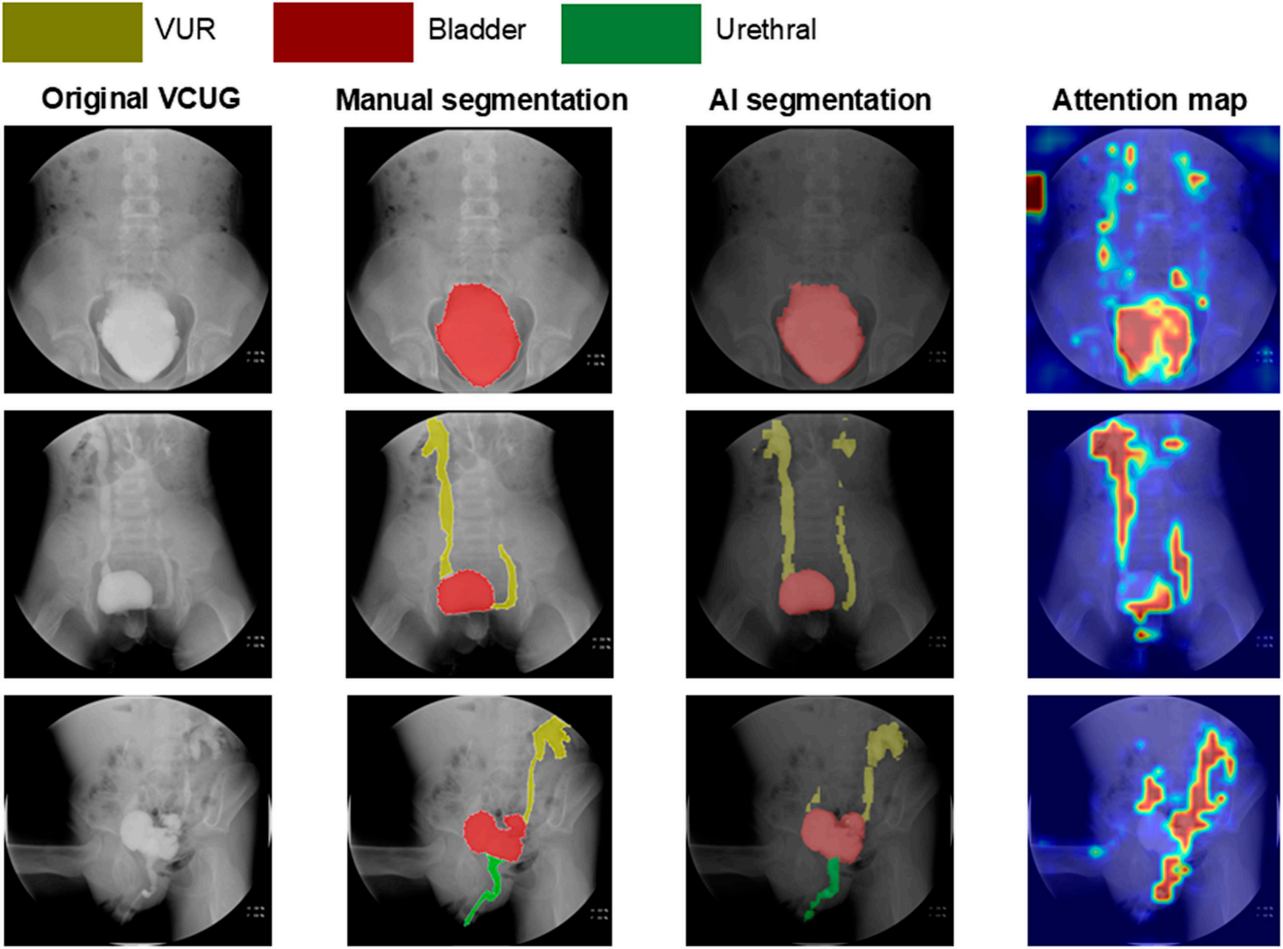
Three VCUG images selected from the testing set (left) with manual segmentation (second column), automated classification and segmentation (third column), and attention map of the prediction model (right). The 3 colors in the predicted label represent 4 classes of image-level prediction including bladder (red), ureter (yellow), and urethra (green). AI, artificial intelligence.

At the image level, VCUG-DAM generated segmentation maps for lesion regions in each VCUG image (representative examples are presented in Fig. 4). The model demonstrated clearer boundaries and smoother lesions than those of manual segmentation. Additionally, the attention map of the model was analyzed, revealing highlighted pixels that were crucial for distinguishing lesions from background regions (Fig. 4). This indicates that the model effectively learned to focus on the relevant areas of interest during predictions.

### Example of the VCUG-DAM model for patient-level diagnoses

**Fig. 5.**
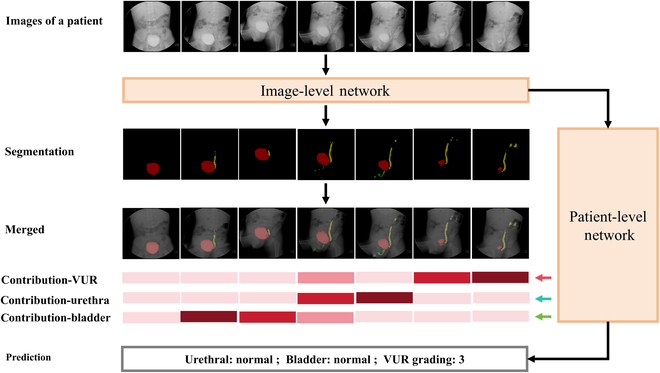
Image weight map of a patient with VUCG examination shown as an example. The first row (raw photos) shows the original VCUG image of a patient. The second row shows the segmentation and diagnosis of a single image of the VCUG-DAM model. The third row (ground truth) shows the segmentation of the VUR, urethra, and bladder on each VCUG image. The fourth row shows the results of merging the segmentation on the original VCUG images. The fifth row shows the calculated contributions to the VUR, urethra, and bladder for each VCUG image. The final row shows the prediction of VCUG-DAM on the patient level.

A patient had more than 2 VCUG images. VCUG-DAM can provide a weight map score for each VCUG image of a patient to indicate the salience. An example of an image attention map is shown in Fig. 5. The image weight map demonstrates how VCUG-DAM automatically assigned greater weights to the bladder, urethra, and ureters, reflecting its focus on lesion areas. This feature aids clinicians by highlighting critical slices and providing insights into how the model prioritizes specific regions within the images.

## Discussion

In this study, we developed and tested VCUG-DAM, an AI model for segmenting and diagnosing ureter, bladder, and VUR conditions using VCUG images at both image and patient levels. The model alone achieved a high diagnostic performance, surpassing that of clinicians. Furthermore, segmentation results and slice attention maps offered valuable tools for visualizing and locating lesion regions, aiding clinician diagnoses.

VCUG remains the gold standard for diagnosing various urological conditions, such as diverticula, trabeculation, ureterocele, posterior urethral valve, anterior urethral valve, and urethral stenosis. Owing to the invasive nature of VCUG, alternatives such as ceVUS have been proposed for VUR grade [15]; however, it cannot present panoramic views, leading to the inability to visualize the urethra and reno-ureteral system in one view during voiding [16–18].

The international reflux study in 1985 published the first reflux protocol and graded VUR into 5 classes [19]. However, this VUR grading has poor interobserver reliability. Some objective and reliable parameters including the distal ureteral diameter ratio, renal cross-sectional area, pelvis dilatation, and tortuosity predictors of VUR grade even predicted the frequency of urinary tract infections [20,21]. Additionally, several AI-assisted tools have been developed to grade VUR using VCUG images [13,14,22,23]. For example, Khondker et al. [22,23] developed a machine learning-based model, qVUR, which enables users to extract metrics such as the ureteropelvic junction width, ureterovesical junction width, maximum ureter width, and ureter tortuosity from an uploaded VCUG image. This model utilizes a random forest classifier trained to distinguish between low- and high-grade VUR. In addition, Eroglu et al. [13] proposed a CNN-based model for grading VUR from VCUG images. However, this model is not suitable for bilateral VUR grading and has limitations, including a small sample size and a single-center design. Chen et al. [24] used the Dual Stream CNN Model for VUR grading. Another study by Li et al. [14] employed an ensemble learning method for grading VUR, which was validated in a single-center study. Despite these advancements, all existing VCUG-based AI tools for VUR grading operate at the image level and rely on manually extracted slices, requiring experienced clinicians for interpretation. None of these tools are designed for the clinical grading of VUR at the patient level. The VCUG-DAM is a patient-level diagnostic system that extracts information from multiple VCUG images and assigns weights to generate a diagnosis for a single patient. This approach eliminates the need for manual slice selection and enhances diagnostic accuracy. Under VCUG-DAM assistance, both the accuracy and consistency of VUR grading significantly improve.

Additionally, 3 previous studies have reported on the diagnosis of bladder or urethral abnormalities using the DL method. Kim et al. [25] proposed a DL model to differentiate urethral strictures from normal slices. Abdovic et al. [26] developed a prediction model for late-presenting posterior urethral valves in boys with lower urinary tract symptoms using an artificial neural network. Their findings highlighted the potential of AI-assisted diagnosis for echinococcal urethral strictures. Weaver et al. [27] applied a DL method to classify the severity of bladder dysfunction in patients with spina bifida using video urodynamics. VCUG remains the gold standard for diagnosing urethral strictures. Our study revealed that the VCUG-DAM effectively assessed normal and abnormal bladders and ureters for conditions such as diverticula, trabeculations, ureterocele, posterior urethral valve, anterior urethral valve, and urethral stenosis.

The AI model has several advantages. It performs classification and segmentation tasks, accurately diagnoses VUR, and distinguishes between bladder or urethral abnormalities. Moreover, it can be used to segment lesions in the bladder, urethra, and ureter. A typical VCUG examination involves multiple images that reflect the morphology of the bladder, urethra, and ureter at different stages. Furthermore, the contribution of each image to the final diagnosis varies. To address this, the model incorporates 2 important design features. First, it stacks high-dimensional features extracted from multiple images, capturing morphological changes in the bladder, urethra, and ureters across different stages of the examination. Second, we introduced disease-specific learnable category embeddings that automatically determine the contribution of each image to the diagnosis of various conditions, thereby reducing the need for manual intervention.

Additionally, our AI model has great potential to improve the diagnostic performance of clinicians. We conducted a multi-reader, multi-case study using an AI diagnosis for VCUG. The results indicated a diagnostic improvement in patient-level analysis when employing the AI-assisted strategy. Resident clinicians achieved the highest improvement with AI assistance, approaching the diagnostic level of senior clinicians. Additionally, the model assisted in improving the consistency of VUR grading. Hence, the AI model can be used as a frontline tool in remote or low-income regions with scarce medical resources.

Our AI model involved a multicenter study of VCUG images. To our knowledge, this is the largest sample of patients to date. Data were collected from 15 centers, including different hospitals across China. We used internal and external validation dataset 1 to test the diagnostic ability of the model on these datasets and external validation dataset 2 to assess VUR grading. The inclusion of data from multiple institutions helps to minimize potential biases associated with single-center studies and increases the clinical applicability of the model [10,28].

Furthermore, the AI model can visualize localized lesions. Our model segmentation showed clear and smooth boundaries for the lesions. An attention map of the image-level model was also examined, which highlighted the pixels relevant to differentiating these lesions from background fields, suggesting that the model generally learned to focus on the areas of interest when making predictions. Together with the slice attention map, the segmentation map assisted clinicians in locating and visualizing lesion regions [29,30].

However, this study has some limitations. First, the ground truth was determined by expert readings, which, while reliable, could vary between clinicians. To address this, diagnostic labels were assigned through independent assessment by 2 experts, with consensus discussions and third-party adjudication when needed. Second, the dataset had an insufficient number of normal bladder and urethral samples. To address this, we used a model involving a multitasking learning strategy based on a Vision Transformer (ViT) architecture [31,32]. The class token from ViT was used to predict tasks, such as bladder and urethral abnormalities and bilateral VUR grading. Image tokens were employed for a confidence-based semantic task, with attention maps from both tokens guiding the segmentation. This approach improved the overall performance and effectively mitigated the impact of data imbalance, facilitating generalization and more reliable predictions. Third, although a 4-week washout period and randomized ordering of VCUG cases were applied in the multi-reader multi-case study, recall bias could not be eliminated. Fourth, this study did not perform detailed comparisons of discordance between VCUG-DAM and clinicians. Future research will address this to further validate the tool’s clinical applicability. Last, prospective studies with larger sample sizes were lacking. We look forward to testing and refining the VCUG-DAM in prospective studies with larger populations.

## Conclusion

We developed an AI model, VCUG-DAM, to segment and diagnose VCUG at the imaging and patient levels more accurately than experienced clinicians. Our approach can be applied in clinical settings by assisting clinicians in diagnosing and segmenting lesion regions.

## Methods

### Data collection

**Fig. 6.**
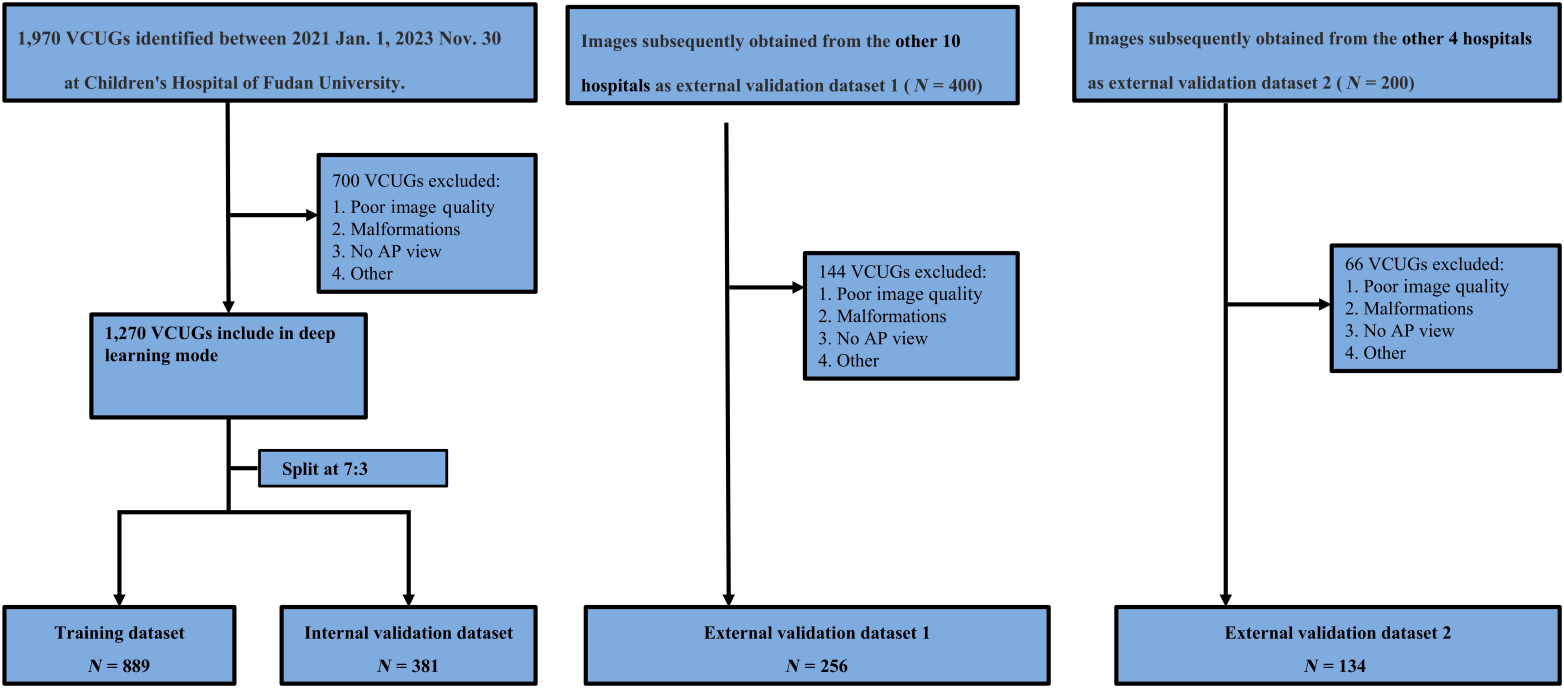
Flowchart of the study population.

A flowchart of the data collection process is shown in Fig. 6. The study included participants who were 18 years of age or younger; had been diagnosed with urethral abnormalities, bladder abnormalities, vesicoureteral reflux (VUR), or had normal VCUG findings; had complete medical records and examination data; and had clear, high-quality imaging available for analysis.Exclusion criteria included data loss, incomplete data, or poor-quality VCUG images containing severe artifacts. Detailed inclusion and exclusion criteria are shown in Appendix S1 and Figs. S1 and S2. A total of 1,660 VCUG were retrospectively collected from 15 hospitals in China: the Children’s Hospital of Fudan University was used for VCUG-DAM training and internal validation; 10 hospitals comprised external validation dataset 1, and another 4 hospitals comprised external validation dataset 2.

### Ground-truth development

To provide ground-truth classification and segmentation for training and testing VCUG-DAM, a total of 1,660 VCUG examinations, which contained 7,899 VCUG images, were delineated by 3 clinicians (with 5 years of experience in VCUG interpretation) using the LabelMe software. At least 2 experts delineated each image. The ground truth was determined based on the overlapping areas between the delineations of the 2 experts. The ground truth of patient diagnosis was based on multi-VCUG images. If the results were consistent, the label would be adopted. However, if the results were discordant, another experienced pediatric radiologist (with 20 years of experience in pediatric radiology interpretation) would review and check the discrepancy and determine the gold standard result. The details of the delineation process for the VCUG images are described in the appendix method (Appendix S1).

### VCUG-DAM development

**Fig. 7.**
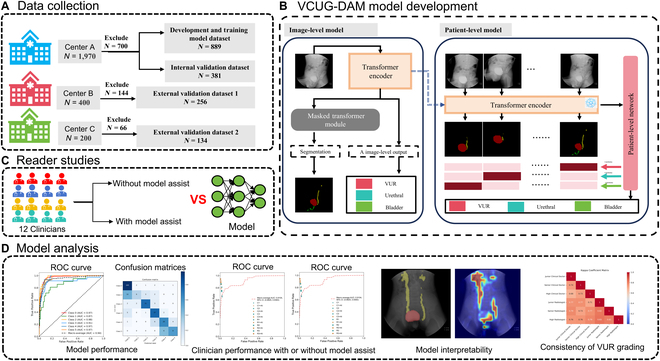
Overview of the study design. (A) Data collection. (B) Development of VCUG-DAM models for the image level and patient level on the VCUG image. (C) Twelve clinicians without and with the assistance of VCUG-DAM to diagnosis. (D) Model analysis. Center A is Children’s Hospital of Fudan University; Center B consists of 10 hospitals; Center C consists of 4 hospitals.

The architecture of VCUG-DAM consists of 2 main models: the image-level model and the patient-level model (Fig. 7B). The image-level model simultaneously handles classification and segmentation tasks using a unified transformer-based architecture. The model takes an input image and outputs lesion masks for the bladder, urethra, and ureter, along with the predicted lesion grade, including bladder abnormalities, urethral abnormalities, and L- and R-VUR grades. The model is initialized with ViT-Base weights pre-trained on ImageNet using masked self-supervision, which stabilizes training and accelerates convergence, enhancing the representation capability of the image-level model.

The patient-level model uses the weights from the image-level model as its initial weights, which are frozen to extract high-dimensional feature representations for each image. These disease-relevant features are stacked into a matrix, and a multitask attention layer is then designed to dynamically assess the contribution of each high-dimensional representation. This enables the model to generate a final prediction on whether a patient has bladder or urethral abnormalities and to determine the grade of ureteral reflux.

The detailed implementation of the image-level and patient-level models can be found in Appendix S2.

### Study design

A dataset of 1,270 VCUG with 5,644 VCUG images was collected for training and validation of VCUG-DAM, from the Children’s Hospital of Fudan University. Using a non-overlap patient-level splitting with a ratio of 7:3, patients were randomly divided into the training dataset and the internal validation dataset. For stage 1, we adopted the internal validation dataset to assess the image-level and patient-level classification performance of the VCUG-DAM model.

For stage 2, in external validation dataset 1, we assessed the patient-level diagnosis performance of the VCUG-DAM model. In addition, we explored whether the VCUG-DAM model could improve the diagnostic ability of clinicians. Twelve clinicians with varying levels of experience (i.e., junior, attending, and senior clinicians) were recruited. First, 12 clinicians assessed the results of VUR, bladder, and ureter based on VCUG. Then, a crossover design was used with a washout period of at least 4 weeks. Twelve clinicians reassessed the results based on the predicted probability of results provided by VCUG-DAM after 4 weeks. They could choose either not to change or to just read the initial results.

For stage 3, external validation dataset 2 was used to evaluate whether the use of the VCUG-DAM model could improve the consistency of patient-level VUR grading among clinicians. First, 12 clinicians assessed the grading of VUR according to International Reflux Society classification criteria [33]. Then, they reassessed the VUR grade based on the predicted probability provided by VCUG-DAM after 4 weeks. They could choose either not to change or to just read the initial results. The agreement between all pairs of clinicians with and without VCUG-DAM assistance was calculated using Cohen’s kappa value.

All experiments were done in accordance with the Declaration of Helsinki and reported in adherence with the Standards for Reporting Diagnostic Accuracy. This study was approved by the Institutional Review Board (IRB) of the Children’s Hospital of Fudan University [approval no. 2024(46)]. As this was a retrospective study using anonymized data, and no patient contact or intervention was involved, additional IRB approvals from the other participating hospitals were not required in accordance with local regulations and institutional policies. It was also registered in the Chinese Clinical Trial Registry (registration number: ChiCTR2400088035)

### Statistical analysis

We evaluated the image-level and patient-level performance using 5 metrics: area under the receiver operating characteristic curve (AUROC), accuracy, sensitivity, specificity, and F1 score. The AUROC, sensitivity, specificity, and accuracy of the test sets were calculated and presented with 95% CIs. Moreover, a confusion matrix was used to assess the image-level and patient-level model performance for each classification task. Two-sided *t* tests were conducted to identify significant differences. For bladder or urethral abnormalities tasks, the AUROC, sensitivity, specificity, and F1 score were calculated in a binary setting. For L- and R-VUR grade tasks, the AUROC, sensitivity, specificity, and F1 score were calculated for each disease category and then averaged to obtain the final metric value. A minimum sample size of 85 was determined to be sufficient to detect a mean AUC difference of 0.05 in the diagnostic accuracy of the 12 participating readers (alpha = 0.05, beta = 0.20) (Appendix S3).

All statistical analyses were conducted using the following software and packages: Scikit-learn (version 1.3.2), Torchvision (version 0.10.1), SciPy (version 1.10.1), Python (version 3.8.2), PyCharm (version 4.0), Timm (version 0.5.4), Seaborn (version 0.11.2), OpenCV-python (version 4.9.0.80), and Matplotlib (version 3.7.5).

## Data Availability

All data collected for the study are not publicly available for download due to patient confidentiality and consent. However, the corresponding authors can be contacted for academic inquiry. The source code for the deep learning model is available through https://github.com/wangzheyuan-666/VCUG-DAM.git

## Supplementary Materials

Supplementary 1 Appendices S1 to S3 Figs. S1 and S2 Tables S1 to S11
