# Supplementary material for: Dynamic Multi-Image Weighting for Automated Detection and Diagnosis of Abnormal Urinary Tract on Voiding Cystourethrography with a Deep Learning System: A Retrospective, Large-Scale, Multicenter Study
Source: Research (Wash D C). 2025 Jul 22;8:0771. doi: 10.34133/research.0771 (PMC12280329; doi:10.34133/research.0771)
Supplement: Supplementary 1 — Appendices S1 to S3 Figs. S1 and S2 Tables S1 to S11 [file research.0771.f1.docx]

**Supplementary** **Appendix**

**Contents**

| **Supplementary item** |
| --- |
| **Study method** |
| Supplemental S1 |
| Supplemental S2 |
| Supplemental S3 |
| **Supplementary figures** |
| Figure S1 |
| Figure S2 |
| **Supplementary tables** |
| Table S1 |
| Table S2 |
| Table S3 |
| Table S4 |
| Table S5 |
| Table S6 |
| Table S7 |
| Table S8 |
| Table S9 |
| Table S10 |
| Table S11 |
| **TRIPOD check list** |

**Supplemental** **S1**

**1.1 Inclusion and exclusion criteria of the dataset**

Imaging data of children undergoing VCUG examination due to discomfort such as urinary tract infections. According to the standard protocol for the voiding cystourethrography^[1]^, Ureter Abnormalities (UA), which refers to vesicoureteral reflux (VUR). VUR grading according to the 1958 classification of VUR; Urethral Abnormalities (UA) are included in the inclusion criteria: dilatation, valves, strictures, and the appearance in the region of the external sphincter in the voiding phase. Bladder abnormalities (BA) are included in the inclusion criteria: Filling defects, valves, strictures, and the appearance in the region of the external sphincter. Inclusion criteria for Urethral Abnormalities (UA): dilatation, valves, strictures, and the appearance in the region of the external sphincter during the voiding phase. trabeculations, or Shape and contour abnormalities.

Exclusion criteria were as follows: data loss, incomplete data or poor-quality VCUG images contains severe artifacts (eg, motion artifacts and speed propagation and refraction artifacts) or low image resolution. Figure S1 shows the detailed numbers of included and excluded VCUG images. We retained VCUG images and demographic information including age and sex. We kept more than one VCUG image per patient.

**1.2 Training and internal validation datasets**

VCUG images from the Children's Hospital of Fudan University were combined for the development and internal testing including a total of 1270 voiding cystourethrography, with 5,644 VCUG images. The patients were randomly assigned to a developing set (a training set (70%) and an internal testing set (30%). The developing set contains 889 voiding cystourethrography, with 3,950 VCUG images. The internal validation dataset contains 381 voiding cystourethrography, with 1,694 VCUG images.

**1.3 External validation dataset**

For model external validation, a total of 390 voiding cystourethrography in DICOM format were extracted from 14 hospitals across China. Each VCUG examination consisted of more than 2 VCUG images. The external validation dataset 1 consists of 256 voiding cystourethrography extracted from 10 hospitals(The Six Affiliated Hospital of Harbin Medical University(SHHBMU), The First Affiliated Hospital of Henan University of Traditional Chinese Medicine (FHHN), The Third BETHUNE Hospital of Jilin University(TBHJLU), Jiangxi Provincial Children's Hospital(JXPCH), Xiamen Children's Hospital(XMCH), The First Affiliated Hospital of Xinxiang Medical University(FHXXMU), Honghezhou Maternal and Child Health Hospital(HHZMCH), The First People's Hospital of ZunYi(FPHZY), Guangdong Women and Children Hospital(GWCH), and Wuxi Children's Hospital(WCH)). The dataset 1 including a total of 256 voiding cystourethrography, with 1,765 VCUG images.

The external validation dataset 2 consists of 134 voiding cystourethrography extracted from 4 hospitals (Puyang People’s Hospital; Anhui Provincial Children's Hospital; The Children's Hospital of Guangxi Zhuang Autonomous Region; The Affiliated Hospital of Qingdao University). The dataset 2 including a total of 134 voiding cystourethrography, with 490 VCUG images.

**1.4. Tiered image annotating system**

All VCUG images extracted from the VCUG imaging database were converted into a JPEG format. Image quality control was done for the training set and test sets. For the quality control of VCUG images, all VCUG images were screened and low-quality images containing severe artifacts or significant image resolution reductions were removed. The screening for the images was done by two clinicians (XR, and MW) who had at least 1 year of diagnosis VCUG experience.

For segmentation and classification labeling of the each VCUG image, 3 clinicians (LZC, WM, and LKZ) with more than 5-year working diagnosis VCUG experience manually marked the contour of the ureter, urethral, and bladder lesions of enrolled VCUG images using an annotation software named Labelme. All of markers were reviewed online by experts with 15-year clinical experience (WJT). The markers were revised when any expert thought it inaccurate.

Each patient contains more than two VCUG image. For the label of each patient based on multiple VCUG images. Each patient would be reviewed by two experts (WJT and BJW) with 15 years of experience in VCUG interpretation independently. If the results were consistent, the label would be adopted. However, if the results were discordant, another experienced pediatric radiologist YDL (with 20 years of experience in pediatric radiology interpretation) would review and check the discrepancy and make the gold-standard result.

**Supplemental S2 Details in VCUG-DAM development**

**Image-level Classification and Segmentation**

We propose a unified Transformer-based architecture that simultaneously performs multi-label classification and semantic segmentation, aiming to enhance overall performance through the complementary nature of these tasks. To stabilize training and improve feature representation, we initialize our model with a ViT-Base pre-trained on the ImageNet dataset using masked self-supervised learning. This pre-trained initialization accelerates model convergence and improves the performance of both classification and segmentation tasks.

Our model first encodes the input multi-label images into classification tokens and patch tokens. After processing through the Transformer encoder, the classification tokens are fed into a fully connected layer to perform the multi-label classification task. This classification task is divided into four subtasks: bladder abnormalities, urethral abnormalities, left ureteral reflux grade, and right ureteral reflux grade. To predict the specific label for each subtask, we designed a fully connected layer that maps the classification head into a 16-dimensional feature vector, where different sections of the vector correspond to specific subtask categories: T[0:2] is used to predict bladder abnormalities, T[2:4] predicts urethral abnormalities, T[4:10] predicts left ureteral reflux grade, and T[10:16] predicts right ureteral reflux grade.

While training the classification tasks, we simultaneously designed a masked Transformer module to perform semantic segmentation, specifically targeting the bladder, ureter, and urethra. To further enhance the interaction between the classification and segmentation tasks, we incorporated a self-attention module to facilitate communication between the two. The semantic information from the classification tasks helps the segmentation module better locate regions of interest, while the segmentation tasks, in turn, provide semantic features that assist with classification. This design promotes information sharing between tasks, generating more expressive high-dimensional feature representations.

Next, we detail the encoder and decoder components of the model.

**Encoder**

Given an input image $\mathbf{x}\in\mathbb{R}^{H\times W\times C}$, it is first divided into several patches, represented as $\mathbf{x}=\left[ x_{1},\ldots,x_{N} \right]\in\mathbb{R}^{N\times P^{2}\times C}$, where $(P,P)$ is the patch size, $N=HW/P^{2}$ is the number of patches, and $C$ is the number of image channels. These flattened patches are concatenated with a classification token and augmented with positional encodings, then fed into a Transformer encoder consisting of $L$ layers to generate high-dimensional representations. Each layer comprises a multi-head self-attention (MSA) module and a multi-layer perceptron (MLP) module.

Finally, we use an MLP head to map the classification token to the final disease predictions, which include bladder abnormalities, urethral abnormalities, left ureteral reflux grade, and right ureteral reflux grade. Among these, bladder and urethral abnormalities are binary classification tasks, while the ureteral reflux grading consists of six levels, where level 0 represents normal. Thus, the classification head outputs a 16-dimensional feature vector that distinguishes the disease categories of interest. During training, these four subtasks are processed simultaneously.

**Decoder**

While training the classification tasks, we concurrently perform segmentation tasks, which include segmenting the bladder, ureter, and urethra. At this stage, the patch and classification token sequences are passed through two self-attention blocks to generate class and semantic representations. A multi-task attention layer is designed to process these representations at the matrix level, yielding a clue-based mask that reflects the contribution of each patch. The up sampled semantic representation is resized to the original image dimensions using the “pixel shuffle” function. After performing element-wise multiplication with the clue mask, the final segmentation map $\mathbf{s}\in\mathbb{R}^{H\times W\times K}$ is obtained, where $K$ is the number of categories.

**Patient-Level Classification**

After achieving classification at the image level, we extend the model to perform instance-level classification, aiming to automatically identify the contribution of each image in a set of patient images and produce a final classification. The process is as follows:

First, we load the pre-trained weights from the image-level encoder and freeze these parameters, denoted as $T_{\text{frozen}}$. We set the maximum number of images per patient to 10. For patients with fewer than 10 images, padding with zero-valued images ensures that the input size remains consistent. These images are then sequentially input into the frozen classifier $T_{\text{frozen}}$, extracting high-dimensional feature representations. The features from all images are stacked into a matrix, denoted as $\mathbf{H}\in\mathbb{R}^{10\times D}$, where $D$ represents the feature dimension.

To perform instance-level classification, we introduce a set of $K$ learnable class embeddings, $\text{cls}=[\mathrm{cls}_{1},\ldots,\mathrm{cls}_{K}]\in\mathbb{R}^{K\times D}$, where $K=3$, corresponding to the tasks of bladder abnormalities, urethral abnormalities, and ureteral reflux grading. Each class embedding, along with the high-dimensional feature matrix $\mathbf{H}$ from the 10 images, is processed through the decoder. Finally, we use three independent classification heads, where each head maps a class embedding to a probability distribution over the possible disease categories. This architecture enables the model to automatically learn the contribution of each image to the classification task for each disease.

**Supplemental S3**

**3.1 Sample size calculation**

We adopted the conjectured parameter for a nonequivalence test (alpha=0.05, beta=0.80), treating both the readers and cases as random. We assumed that the mean receiver operating characteristic curve (AUC) between tests with and without the AI model was no lower than 0.70. We expected that the variability among readers’ comparisons of the two tests, i.e., the test1 – test2 true AUC differences was within a range no larger than 0.05. We chose a conservative conjectured value of 0.80 for r1, and 0.75 as a conservative conjectured value for r2 - r3. Results show that 12 readers (each subgroup of clinicians in our study) were needed for 85 cases.

**3.2 Clinical readers recruitment**

A total of 12 certified clinical readers in this study were recruited from 3 hospitals across China, including four resident clinicians (2 pediatric radiologists and 2 pediatric urologists), four attending clinicians (2 pediatric radiologists and 2 pediatric urologists), and four senior clinicians (2 pediatric radiologists and 2 pediatric urologists). The recruitment criteria were as follows: 1) resident radiologists: more than two years of work experience with at least one year of experience in VCUG interpretation; 2) attending radiologists: more than five years of work experience with at least three years of experience in VCUG interpretation; 3) senior radiologists: more than ten years of work experience with at least five years of experience in VCUG interpretation; 4) resident Pediatric urologist: more than two years of work experience; 5) attending Pediatric urologist: more than five years of work experience

**Reference**

[1] Janssen K M, Kirsch A J, Crisostomo-Wynne T C, Leong T, Cuda S P, Arlen A M. Standardized protocol for voiding cystourethrogram: Are recommendations being followed? [J]. Journal of pediatric urology, 2021, 17(1): 66.e61-66.e66.

**Supplementary Figures**

**Figure S1 Workflow diagram**

| 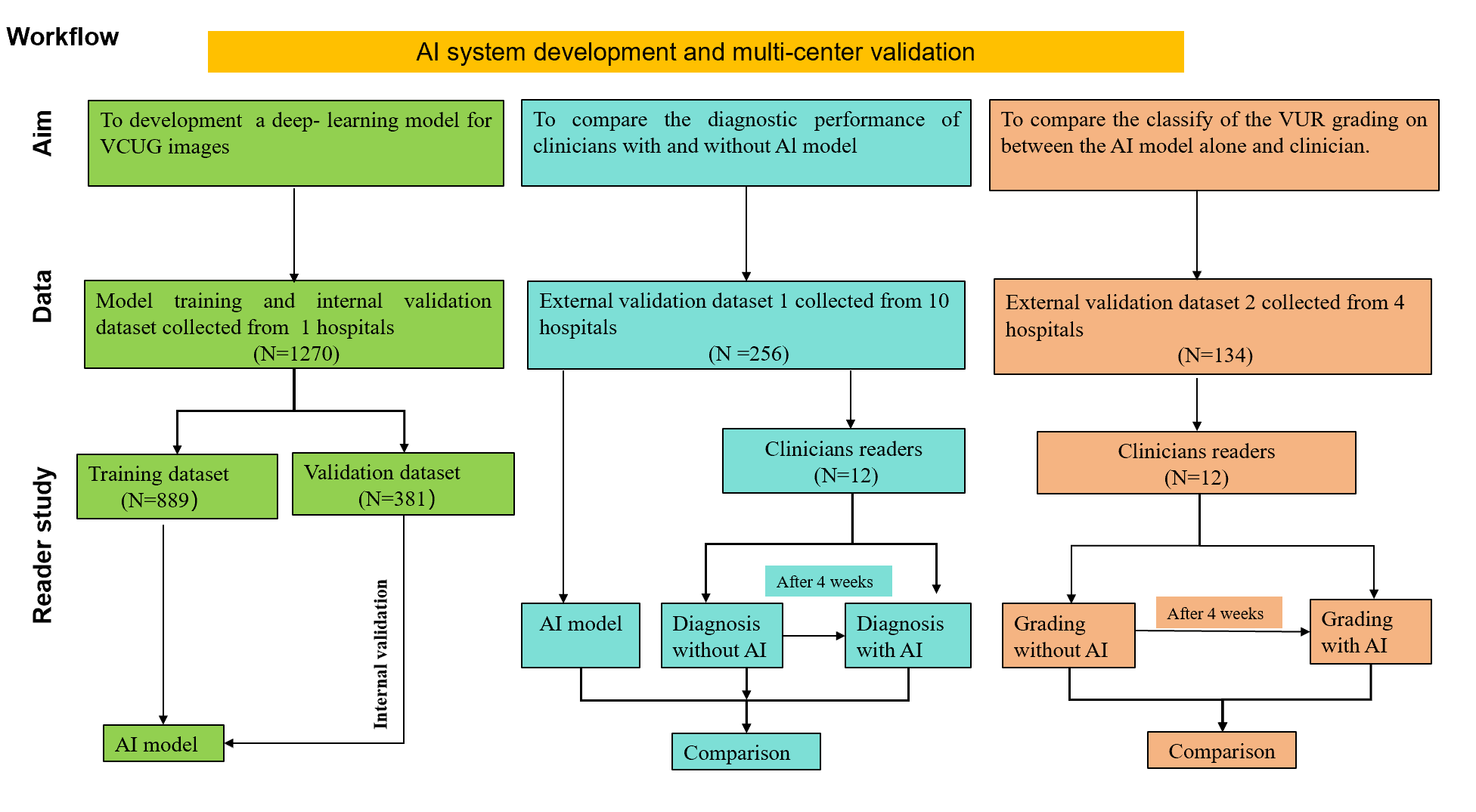 |
| --- |
| **AI=artificial intelligence; CHFU= Children's Hospital of Fudan University** |

**Figures S2****: Flowchart of the training and external validation dataset**

| **1970 patients with from CHFU**  **(****Jan 1, 2021 to Nov 30, 2023)**  **Patients Exclude**  **700 Data loss/unqualified VCUG image**  **CHFU cohort**  **(N= 1270)** |  | **45 patients with from** **PYPH**  **(Jan 1, 2021 to** **Nov 30, 2023)**  **Patients Exclude**  **25 Data loss/unqualified VCUG image**  **PYPH cohort**  **(N= 20)**  **51 patients with from APCH**  **(Jan 1, 2021 to Nov 30, 2023)**  **Patients Exclude**  **15 Data loss/unqualified VCUG image**  **APCH cohort**  **(N=36)** |
| --- | --- | --- |
| **37 patients with from** **CHGXZAR**  **(Jan 1, 2021 to Nov 30, 2023)**  **Patients Exclude**  **6 Data loss/unqualified VCUG image**  **CHGXZAR cohort**  **(N= 31)** | **67 patients with from AHQU**  **(Jan 1, 2021 to Nov 30, 2023)**  **Patients Exclude**  **20 Data loss/unqualified VCUG image**  **AHQU cohort**  **(N= 47)** | **68 patients with from SHHBMU**  **(Jan 1, 2021 to Nov 30, 2023)**  **Patients Exclude**  **20 Data loss/unqualified VCUG image**  **SHHBMU cohort**  **(N=68)** |
| **7 patients with from** **FHHN**  **(Jan 1, 2021 to Nov 30, 2024)**  **Patients Exclude**  **4 Data loss/unqualified VCUG image**  **FHHN cohort**  **(N=3)** | **7 patients with from TBHJLU**  **(Jan 1, 2021 to Nov 30, 2024)**  **Patients Exclude**  **5 Data loss/unqualified VCUG image**  **TBHJLU cohort**  **(N=2)** | **78 patients with from JXPCH**  **(Jan 1, 2021 to Nov 30, 2024)**  **Patients Exclude**  **34 Data loss/unqualified VCUG image**  **JXPCH cohort**  **(N=44)** |
| **22 patients with from XMCH**  **(Jan 1, 2021 to Nov 30, 2024)**  **Patients Exclude**  **10 Data loss/unqualified VCUG image**  **XMCH cohort**  **(N=12)**  **10 patients with from FHXXMU**  **(Jan 1, 2021 to Nov 30, 2024)**  **Patients Exclude**  **6 Data loss/unqualified VCUG image**  **FHXXMU cohort**  **(N=4)**  **10 patients with from HHZMCH**  **(Jan 1, 2021 to Nov 30, 2024)**  **Patients Exclude**  **5 Data loss/unqualified VCUG image**  **HHZMCH cohort**  **(N=5)** |  |  |
| **16 patients with from FPHYZ**  **(Jan 1, 2021 to Nov 30, 2024)**  **Patients Exclude**  **10 Data loss/unqualified VCUG image**  **FPHYZ cohort**  **(N=6)** | **54 patients with from CWCH**  **(Jan 1, 2021 to Nov 30, 2024)**  **Patients Exclude**  **20 Data loss/unqualified VCUG image**  **CWCH cohort**  **(N=34)** | **108patients with from WCH**  **(Jan 1, 2021 to Nov 30, 2024)**  **Patients Exclude**  **30 Data loss/unqualified VCUG image**  **WCH cohort**  **(N=78)** |
| **Flowchart of the dataset.**  CHFU=Children's Hospital of Fudan University；PYPH= Puyang People’s Hospital; APCH= Anhui Provincial Children's Hospital；  CHGXZAR=The Children's Hospital of Guangxi Zhuang Autonomous Region; AHQU= The Affiliated Hospital of Qingdao University; SHHBMU= The Six Affiliated Hospital of Harbin Medical University;  FHHN=The First Affiliated Hospital of Henan University of Traditional Chinese Medicine; TBHJLU=The Third BETHUNE Hospital of Jilin University; JXPCH= Jiangxi Provincial Children's Hospital;  XMCH=Xiamen Children's Hospital; FHXXMU=The First Affiliated Hospital of Xinxiang Medical University; HHZMCH= Honghezhou Maternal and Child Health Hospital;  FPHZY=The First People's Hospital of ZunYi; GWCH=Guangdong Women and Children Hospital; WCH= Wuxi Children's Hospital. | | |

**Supplementary Tables**

**Table S1: Standardized Reporting of Deep Learning Applications in Urology (STREAM-URO) framework.**

| **Methods** | |
| --- | --- |
| **1. Title** | Detection and diagnosis of abnormal urinary tract from Voiding Cystourethrogram images with a deep learning system in China: a retrospective, large-scale, multicentrer study |
| **2. Background and objective** | Voiding cystourethrogram (VCUG) is the gold-standard for delineating the anatomy and determining the function of the lower urogenital tract. Our study aimed to develop a Voiding cystourethrogram Diagnostic AI system (VCUG-DAM) for segmentation and diagnosis the bladder, urethral, and VUR grading based on VCUG. And, assess how it helps clinicians improve diagnostic performance and validate its effectiveness in clinical settings. |
| **3. Problem** | All existing VCUG-based AI tools in VUR grading are slice-level prediction models, which take manually extracted slice images. |
| **Source of data** | 1.Department of Urology- Children's Hospital of Fudan University- China Shanghai.  2.Department of Urology- The Six Affiliated Hospital of Harbin Medical University-China. (SHHBMU)  3.Department of Urology- The First Affiliated Hospital of Henan University of Traditional Chinese Medicine-China. (FHHNU)  4.Department of Urology- The Third BETHUNE Hospital of Jilin University-China. (TBHJLU)  5.Department of Urology- Jiangxi Provincial Children's Hospital-China. (JXPCH)  6.Department of Urology- Xiamen Children's Hospital-China. (XMCH)  7.Department of Urology- the First Affiliated Hospital of Xinxiang Medical University-China. (FHXXMU)  8.Department of Urology- Honghezhou Maternal and Child Health Hospital. (HHZMCH)  9.Department of Urology- the First People's Hospital of Zunyi-China. (FPHZY)  10.Department of Urology- Guangdong Women and Children Hospital-China. (GWCH)  11.Department of Urology- Wuxi Children's Hospital (WCH)  12. Department of Urology- Puyang People’s Hospital- Henan- China  13. Department of Urology- Anhui Provincial Children's Hospital- Anhui- China.  14. Department of Urology- The Children's Hospital of Guangxi Zhuang autonomous region- China.  15. Department of Urology- The Affiliated Hospital of Qingdao University- China. |
| **4. Eligibility Criteria** | See the Supplemental file 1, 1.1 Inclusion and exclusion criteria of the dataset |
| **5. Label** | See the Supplemental file 1, 1.4. Tiered image annotating system |
| **6.** **Data Preparation** | **- Data preparation：**All VCUG images were automatically cropped to a 512 × 512 matrix, as this invariably included the entire bladder and ureter and is the input utilized by our model. Then, all individual scans were normalized to the 0~1 interval by subtracting the mean value and dividing by the variance.  **-Data augmentation** We apply a set of data augmentation techniques to enhance the model's generalization and performance. It includes random affine transformations with rotation, translation, and scaling, random perspective transformations for realistic perspective changes, random sharpness adjustments to vary image sharpness, and random horizontal flips to create mirrored versions of the images. These augmentations introduce diversity into the dataset, allowing the model to better adapt to various scenarios and improve its overall performance. |
| **7. Data splitting** | **Training/ internal Validation dataset**  -Department of Urology- Children’s Hospital of Fudan University- China Shanghai. The participants were split at 889% (N = 1000); 30% (N =381) by using split-sample validation into training and internal testing sets- respectively.  **External validation dataset 1**  -Department of Urology- The Six Affiliated Hospital of Harbin Medical University-China. (SHHBMU)  -Department of Urology- The First Affiliated Hospital of Henan University of Traditional Chinese Medicine-China. (FHHNU)  -Department of Urology- The Third BETHUNE Hospital of Jilin University-China. (TBHJLU)  -Department of Urology- Jiangxi Provincial Children’s Hospital-China. (JXPCH)  -Department of Urology- Xiamen Children’s Hospital-China. (XMCH)  -Department of Urology- the First Affiliated Hospital of Xinxiang Medical University-China. (FHXXMU)  -Department of Urology- Honghezhou Maternal and Child Health Hospital. (HHZMCH)  -Department of Urology- the First People’s Hospital of Zunyi-China. (FPHZY)  -Department of Urology- Guangdong Women and Children Hospital-China. (GWCH)  -Department of Urology- Wuxi Children’s Hospital (WCH)  **External validation dataset 2**  -Department of Urology- Puyang People‘s Hospital- Henan- China. (PYPH)  -Department of Urology- Anhui Provincial Children’s Hospital- Anhui- China. (APCH)  -Department of Urology- The Children’s Hospital of Guangxi Zhuang Autonomous Region- China. (CHGXZAR)  -Department of Urology- The Affiliated Hospital of Qingdao University- China. (AHQU) |
| **8.** **Reference standard** | The model will compare with the 12 clinicians on the external dataset 1 |
| **9.Hyperparameter tuning** | During model training, the batch size is set to 16. The total number of training epochs is 50, with the first 10 epochs dedicated to learning rate warm-up (from 0 to 5e-4). This is followed by a cosine annealing schedule, reducing the learning rate from 5e-4 to 1e-6 over the remaining 40 epochs. |
| **10. Model evaluation** | Accuracy、AUROC、Precision、Sensitivity 、specificity and F1 score |
| **11. Model explanation** | Attention activation maps |

**Table S2 Patient Demographic and Clinical Characteristics**

| Number | Hospital | Type | Patient | Age  (months) | Sex | | Number of VCUG images |
| --- | --- | --- | --- | --- | --- | --- | --- |
|  |  |  |  |  | Male | Female |  |
| 1 | Department of Urology- Children's Hospital of Fudan University- China Shanghai. **(CHFU)** | Model training and internal validation dataset | 1270 | 42.12±40.78 | 780(61.42%) | 490(38.58%) | 5644 |
| 2 | Department of Urology- The Six Affiliated Hospital of Harbin Medical University-China. (**SHHBMU**) | External validation dataset 1  (Multi-reader multi-case study, 12 clinicians) | 68 | 67.46±31.75 | 33 (48.53%） | 35 (51.47%） | 460 |
| 3 | Department of Urology- The First Affiliated Hospital of Henan University of Traditional Chinese Medicine-China. (**FHHNU**) |  | 3 | 48.00±0.00 | 2 (66.67%） | 1 (33.33%） | 15 |
| 4 | Department of Urology- The Third BETHUNE Hospital of Jilin University-China. (**TBHJLU**) |  | 2 | 72.00±36.00 | 2 (100.00%） | 0 (0.00%） | 20 |
| 5 | Department of Urology- Jiangxi Provincial Children's Hospital-China. (**JXPCH**) |  | 44 | 33.89±29.41 | 25 (56.82%） | 19 (43.18%） | 378 |
| 6 | Department of Urology- Xiamen Children's Hospital-China. (**XMCH**) |  | 12 | 27.64±25.82 | 4 (33.33%） | 8 (66.67%） | 70 |
| 7 | Department of Urology- the First Affiliated Hospital of Xinxiang Medical University-China. (**FHXXMU**) |  | 4 | 72.00±24.00 | 3 (75.00%） | 1 (25.00%） | 28 |
| 8 | Department of Urology- Honghezhou Maternal and Child Health Hospital. (**HHZMCH**) |  | 5 | 13.20±14.66 | 3 (60.00%） | 2 (40.00%） | 38 |
| 9 | Department of Urology- the First People's Hospital of Zunyi-China. **(FPHZY**) |  | 6 | 50.44±26.73 | 5 (83.33%） | 1 (16.67%） | 34 |
| 10 | Department of Urology- Guangdong Women and Children Hospital-China. (**GWCH**) |  | 34 | 24.45±30.64 | 22 (64.71%） | 12 (35.29%） | 222 |
| 11 | Department of Urology- Wuxi Children's Hospital (**WCH**) |  | 78 | 38.42±32.58 | 35 (44.87%） | 43 (55.13%） | 500 |
| 12 | Department of Urology- Puyang People’s Hospital- Henan- China.  **(PYPH)** | External validation dataset 2  (Multi-reader multi-case study, VUR grading) | 20 | 41.35±24.95 | 14 (70.00%） | 6 (30.00%） | 116 |
| 13 | Department of Urology- Anhui Provincial Children's Hospital- Anhui- China. **(APCH)** |  | 36 | 41.75±30.24 | 17 (47.22%) | 19 (52.78%） | 89 |
| 14 | Department of Urology- The Children's Hospital of Guangxi Zhuang Autonomous Region- China. **(CHGXZAR)** |  | 31 | 40.06±31.30 | 21(67.74%) | 10 (32.26%) | 134 |
| 15 | Department of Urology- The Affiliated Hospital of Qingdao University- China. **(AHQU)** |  | 47 | 41.87±28.03 | 30 (63.83%) | 17 (36.17%) | 151 |

**Table S3 The performance of the VCUG-DAM for image-level on the internal validation dataset**

|  | **Accuracy**  **(95% CI)** | **AUROC**  **(95% CI)** | **Sensitivity**  **(95% CI)** | **Specificity**  **(95% CI)** | **Precision**  **(95% CI)** | **F1 Score**  **(95% CI)** |
| --- | --- | --- | --- | --- | --- | --- |
| **Bladder** | 0.9442(0.9327,0.9545) | 0.9507(0.9340,0.9650) | 0.6649(0.6000,0.7312) | 0.9824(0.9751,0.9886) | 0.8372(0.7750,0.8951) | \|0.7406(0.6875,0.7910) |
| **Urethral** | 0.9723(0.9640,0.9799) | 0.9408(0.9112,0.9674) | 0.5502(0.4354,0.6667) | 0.9914(0.9863,0.9957) | 0.7414(0.6111,0.8572) | 0.6298(0.5161,0.7242) |
| **L-VUR-Grade** | 0.8868(0.8719,0.9020) | 0.9568(0.9463,0.9662) | 0.6670(0.6216,0.7166) | 0.9623(0.9564,0.9679) | 0.7064(0.6543,0.7606) | 0.6806(0.6360,0.7251) |
| **R-VUR-Grade** | 0.9246(0.9115,0.9368) | 0.9249(0.9006,0.9465) | 0.6340(0.5776,0.6887) | 0.9427(0.9319,0.9529) | 0.7022(0.6373,0.7643) | 0.6514(0.5943,0.7034) |

**Table S4 The performance of the VCUG-DAM for patient-level on the internal validation dataset**

|  | **Accuracy**  **(95% CI)** | **AUROC**  **(95% CI)** | **Sensitivity**  **(95% CI)** | **Specificity**  **(95% CI)** | **Precision**  **(95% CI)** | **F1 Score**  **(95% CI)** |
| --- | --- | --- | --- | --- | --- | --- |
| **Bladder** | 0.8740(0.8399,0.9081) | 0.8772(0.8279,0.9265) | 0.5192(0.4032,0.6351) | 0.9618(0.9402,0.9833) | 0.7758(0.6604,0.8913) | 0.6184(0.5177,0.7190) |
| **Urethral** | 0.8661(0.8320,0.9003) | 0.7752(0.7094,0.8410) | 0.2833(0.1667,0.4000) | 0.9765(0.9594,0.9935) | 0.6903(0.5000,0.8805) | 0.3955(0.2609,0.5301) |
| **L-VUR-Grade** | 0.8570(0.8215,0.8924) | 0.9443(0.9242,0.9644) | 0.6665(0.5937,0.7393) | 0.9599(0.9493,0.9704) | 0.7174(0.6318,0.8030) | 0.6778(0.6032,0.7523) |
| **R-VUR-Grade** | 0.8517(0.8163,0.8871) | 0.9342(0.9065,0.9618) | 0.6728(0.5839,0.7617) | 0.9510(0.9386,0.9635) | 0.7337(0.6262,0.8412) | 0.6666(0.5813,0.7519) |

**Table S5 The performance of the clinicians with and without VCUG-DAM for diagnosis bladder on external validation dataset 1**

|  | **Accuracy**  **(95% CI)** | **AUROC**  **(95% CI)** | **Sensitivity**  **(95% CI)** | **Specificity**  **(95% CI)** | **Precision**  **(95% CI)** | **F1**  **(95% CI)** |
| --- | --- | --- | --- | --- | --- | --- |
| **DL** | 0.9883(0.9805, 0.9961) | 0.9107(0.8584, 0.9629) | 0.8731(0.7917, 0.9545) | 1.0000(1.0000, 1.0000) | 1.0000(1.0000, 1.0000) | 0.9302(0.8837, 0.9767) |
| **Clinicians without AI model** | | | | | | |
| JPU-1 | 0.8184(0.7969,0.8398) | 0.7698(0.7205, 0.8191) | 0.7137(0.6178, 0.8095) | 0.8311(0.8109, 0.8513) | 0.3030(0.2394, 0.3667) | 0.4210(0.3462, 0.4958) |
| JPU-2 | 0.8675(0.8477,0.8873) | 0.8672(0.8307,0.9038) | 0.8620(0.7925, 0.9314) | 0.8679(0.8459, 0.8898) | 0.4021(0.3333, 0.4709) | 0.5504(0.4826, 0.6183) |
| JR-3 | 0.8867(0.8672, 0.9062) | 0.7830(0.7335, 0.8324) | 0.6528(0.5556, 0.7500) | 0.9103(0.8931, 0.9276) | 0.4403(0.3655, 0.5152) | 0.5255(0.4511, 0.6000) |
| JR-4 | 0.9043(0.8828, 0.9258) | 0.8695(0.8283, 0.9108) | 0.8211(0.7388, 0.9035) | 0.9112(0.8913, 0.9311) | 0.4925(0.4183, 0.5668) | 0.6152(0.5455, 0.6849) |
| APU-5 | 0.9066(0.8906, 0.9225) | 0.9118(0.8874, 0.9361) | 0.9137(0.8659, 0.9615) | 0.9057(0.8873, 0.9242) | 0.5105(0.4372, 0.5837) | 0.6577(0.5963, 0.7190) |
| APU-6 | 0.9043(0.8828, 0.9258) | 0.8962(0.8614, 0.9310) | 0.8839(0.8148, 0.9530) | 0.9064(0.8842, 0.9286) | 0.4958(0.4163, 0.5752) | 0.6277(0.5536, 0.7019) |
| AR-7 | 0.8942(0.8783, 0.9102) | 0.8334(0.7942, 0.8725) | 0.7658(0.6915, 0.8400) | 0.9055(0.8879, 0.9231) | 0.4542(0.3939, 0.5146) | 0.5662(0.5083, 0.6241) |
| AR-8 | 0.9098(0.8939, 0.9258) | 0.9145(0.8848, 0.9442) | 0.9147(0.8636, 0.9657) | 0.9067(0.8913, 0.9221) | 0.5092(0.4404, 0.5780) | 0.6512(0.5822, 0.7202) |
| SPU-9 | 0.8812(0.8633, 0.8991) | 0.8945(0.8625, 0.9264) | 0.9059(0.8516, 0.9602) | 0.8796(0.8601, 0.8991) | 0.4422(0.3651, 0.5194) | 0.5860(0.5133, 0.6587) |
| SPU-10 | 0.8948(0.8711, 0.9186) | 0.9073(0.8754, 0.9392) | 0.9109(0.8571, 0.9647) | 0.8940(0.8721, 0.9158) | 0.4694(0.4000, 0.5387) | 0.6205(0.5561, 0.6849) |
| SR-11 | 0.8513(0.8275, 0.8750) | 0.8658(0.8289, 0.9027) | 0.8840(0.8176, 0.9504) | 0.8474(0.8209, 0.8739) | 0.3873(0.3247, 0.4500) | 0.5374(0.4629, 0.6118) |
| SR-12 | 0.8906(0.8711, 0.9102) | 0.9229(0.9032, 0.9427) | 0.9636(0.9272, 1.0000) | 0.8855(0.8639, 0.9071) | 0.4451(0.3670, 0.5232) | 0.6068(0.5327, 0.6808) |
| **Clinicians with AI model** | | | | | | |
| JPU-1 | 0.9121(0.8945, 0.9297) | 0.9143(0.8831, 0.9456) | 0.9114(0.8571, 0.9657) | 0.9130(0.8965, 0.9296) | 0.5157(0.4421, 0.5894) | 0.6542(0.5879, 0.7205) |
| JPU-2 | 0.9297(0.9141, 0.9453) | 0.9406(0.9181, 0.9630) | 0.9577(0.9155, 1.0000) | 0.9259(0.9114, 0.9404) | 0.5781(0.4981, 0.6581) | 0.7156(0.6441, 0.7870) |
| JR-3 | 0.9277(0.9102, 0.9453) | 0.9241(0.8950, 0.9533) | 0.9157(0.8636, 0.9677) | 0.9273(0.9107, 0.9439) | 0.5623(0.4960, 0.6286) | 0.6924(0.6396, 0.7452) |
| JR-4 | 0.9297(0.9141, 0.9453) | 0.9214(0.8866, 0.9562) | 0.9280(0.8560, 1.0000) | 0.9282(0.9134, 0.9431) | 0.5711(0.4857, 0.6565) | 0.6963(0.6181, 0.7744) |
| APU-5 | 0.9316(0.9180, 0.9453) | 0.9623(0.9550, 0.9697) | 1.0000(1.0000, 1.0000) | 0.9247(0.9099, 0.9394) | 0.5756(0.5000, 0.6512) | 0.7277(0.6667, 0.7887) |
| APU-6 | 0.9219(0.9062, 0.9375) | 0.9138(0.8869, 0.9406) | 0.9055(0.8494, 0.9615) | 0.9229(0.9083, 0.9375) | 0.5466(0.4750, 0.6181) | 0.6773(0.6126, 0.7421) |
| AR-7 | 0.9492(0.9336, 0.9648) | 0.9193(0.8872, 0.9513) | 0.8834(0.8120, 0.9549) | 0.9554(0.9406, 0.9702) | 0.6745(0.5915, 0.7576) | 0.7591(0.6976, 0.8207) |
| AR-8 | 0.9492(0.9375, 0.9609) | 0.9719(0.9649, 0.9789) | 1.0000(1.0000, 1.0000) | 0.9439(0.9299, 0.9578) | 0.6382(0.5573, 0.7191) | 0.7761(0.7157, 0.8366) |
| SPU-9 | 0.9391(0.9252, 0.9531) | 0.9292(0.9026, 0.9559) | 0.9151(0.8659, 0.9643) | 0.9394(0.9260, 0.9529) | 0.6134(0.5511, 0.6758) | 0.7366(0.6799, 0.7933) |
| SPU-10 | 0.9414(0.9258, 0.9570) | 0.9301(0.8996, 0.9607) | 0.9087(0.8516, 0.9659) | 0.9439(0.9275, 0.9602) | 0.6349(0.5422, 0.7277) | 0.7468(0.6807, 0.8129) |
| SR-11 | 0.9062(0.8867, 0.9258) | 0.9006(0.8616, 0.9396) | 0.8875(0.8182, 0.9568) | 0.9089(0.8904, 0.9274) | 0.4968(0.4142, 0.5793) | 0.6335(0.5588, 0.7081) |
| SR-12 | 0.9004(0.8828, 0.9180) | 0.9256(0.9026, 0.9486) | 0.9580(0.9161, 1.0000) | 0.8949(0.8759, 0.9139) | 0.4748(0.4084, 0.5412) | 0.6363(0.5751, 0.6975) |

Abbreviations: JPU, Junior Pediatric urologist; SPU, Senior Pediatric urologist; JR, Junior radiologist; SR, Senior radiologist; DL, Deep-VCUG model. *The differences between clinicians and DL model, and the differences among clinicians were compared, *P* values were calculated. **P* < 0.05, Significant difference with DL model.

**Table S6 The performance of the** **VCUG-DAM for diagnosis Urethral on external validation dataset 1**

|  | **Accuracy**  **(95% CI)** | **AUROC**  **(95% CI)** | **Sensitivity**  **(95% CI)** | **Specificity**  **(95% CI)** | **Precision**  **(95% CI)** | **F1**  **(95% CI)** |
| --- | --- | --- | --- | --- | --- | --- |
| **DL** | 0.9707(0.9609, 0.9805) | 0.7769(0.7039, 0.8498) | 0.4295(0.2857, 0.5733) | 1.0000(1.0000, 1.0000) | 1.0000(1.0000, 1.0000) | 0.5866(0.4444, 0.7288) |
| **Clinicians without AI model** | | | | | | |
| JPU-1 | 0.9001(0.8822, 0.9180) | 0.6800(0.6181, 0.7418) | 0.4278(0.3000, 0.5556) | 0.9268(0.9116, 0.9421) | 0.2500(0.1667, 0.3333) | 0.3098(0.2105, 0.4091) |
| JPU-2 | 0.9180(0.8984, 0.9375) | 0.6343(0.5626, 0.7060) | 0.3182(0.1749, 0.4615) | 0.9494(0.9346, 0.9641) | 0.2422(0.1333, 0.3511) | 0.2702(0.1529, 0.3874) |
| JR-3 | 0.9352(0.9173, 0.9531) | 0.6776(0.6177, 0.7375) | 0.3859(0.2718, 0.5000) | 0.9668(0.9543, 0.9794) | 0.3856(0.2712, 0.5000) | 0.3815(0.2857, 0.4772) |
| JR-4 | 0.9336(0.9180, 0.9492) | 0.6716(0.6058, 0.7374) | 0.3750(0.2500, 0.5000) | 0.9669(0.9547, 0.9791) | 0.3859(0.2718, 0.5000) | 0.3716(0.2628, 0.4804) |
| APU-5 | 0.9336(0.9180, 0.9492) | 0.6351(0.5682, 0.7021) | 0.3019(0.1653, 0.4386) | 0.9690(0.9585, 0.9795) | 0.3409(0.1818, 0.5000) | 0.3157(0.1917, 0.4398) |
| APU-6 | 0.9587(0.9447, 0.9727) | 0.7953(0.7254, 0.8652) | 0.6109(0.4717, 0.7500) | 0.9735(0.9634, 0.9837) | 0.5545(0.4167, 0.6923) | 0.5789(0.4604, 0.6973) |
| AR-7 | 0.9548(0.9447, 0.9648) | 0.6513(0.5917, 0.7109) | 0.3146(0.1980, 0.4311) | 0.9893(0.9827, 0.9958) | 0.5875(0.3750, 0.8000) | 0.3975(0.2667, 0.5283) |
| AR-8 | 0.9473(0.9336, 0.9609) | 0.6782(0.6091, 0.7473) | 0.3703(0.2353, 0.5053) | 0.9794(0.9710, 0.9877) | 0.5000(0.3333, 0.6667) | 0.4124(0.2609, 0.5639) |
| SPU-9 | 0.9219(0.9062, 0.9375) | 0.7036(0.6325, 0.7746) | 0.4571(0.3117, 0.6025) | 0.9480(0.9338, 0.9623) | 0.3261(0.2308, 0.4214) | 0.3642(0.2712, 0.4571) |
| SPU-10 | 0.9355(0.9219, 0.9492) | 0.5978(0.5349, 0.6606) | 0.2189(0.1044, 0.3333) | 0.9695(0.9593, 0.9797) | 0.2937(0.1429, 0.4444) | 0.2463(0.1111, 0.3815) |
| SR-11 | 0.9355(0.9219, 0.9492) | 0.7810(0.7181, 0.8438) | 0.6163(0.4958, 0.7368) | 0.9541(0.9419, 0.9664) | 0.4184(0.3000, 0.5369) | 0.4838(0.3704, 0.5972) |
| SR-12 | 0.8884(0.8666, 0.9102) | 0.7850(0.7241, 0.8459) | 0.6688(0.5376, 0.8000) | 0.9019(0.8817, 0.9221) | 0.2718(0.1903, 0.3532) | 0.3826(0.2907, 0.4746) |
| **Clinicians with AI model** | | | | | | |
| JPU-1 | 0.9398(0.9258, 0.9538) | 0.7755(0.7065, 0.8445) | 0.6023(0.4545, 0.7500) | 0.9585(0.9460, 0.9710) | 0.4421(0.3333, 0.5509) | 0.4941(0.3882, 0.6000) |
| JPU-2 | 0.9554(0.9453, 0.9655) | 0.7910(0.7161, 0.8658) | 0.6111(0.4691, 0.7531) | 0.9752(0.9666, 0.9837) | 0.5493(0.4286, 0.6700) | 0.5792(0.4706, 0.6878) |
| JR-3 | 0.9665(0.9564, 0.9766) | 0.7582(0.6784, 0.8381) | 0.5353(0.3831, 0.6875) | 0.9878(0.9797, 0.9959) | 0.6962(0.5323, 0.8600) | 0.5994(0.4706, 0.7282) |
| JR-4 | 0.9629(0.9531, 0.9727) | 0.7969(0.7312, 0.8625) | 0.6143(0.4953, 0.7333) | 0.9810(0.9743, 0.9878) | 0.6389(0.5000, 0.7778) | 0.6136(0.5000, 0.7273) |
| APU-5 | 0.9567(0.9447, 0.9688) | 0.7815(0.7116, 0.8513) | 0.5877(0.4466, 0.7288) | 0.9754(0.9675, 0.9834) | 0.5584(0.4211, 0.6958) | 0.5646(0.4444, 0.6847) |
| APU-6 | 0.9727(0.9648, 0.9805) | 0.8037(0.7285, 0.8789) | 0.6163(0.4658, 0.7668) | 0.9918(0.9876, 0.9960) | 0.8093(0.6947, 0.9240) | 0.6857(0.5714, 0.8000) |
| AR-7 | 0.9688(0.9570, 0.9805) | 0.8046(0.7405, 0.8687) | 0.6250(0.5000, 0.7500) | 0.9877(0.9796, 0.9958) | 0.7202(0.5833, 0.8571) | 0.6703(0.5549, 0.7857) |
| AR-8 | 0.9688(0.9570, 0.9805) | 0.7839(0.7182, 0.8496) | 0.5786(0.4529, 0.7044) | 0.9893(0.9827, 0.9959) | 0.7293(0.5814, 0.8772) | 0.6211(0.5000, 0.7422) |
| SPU-9 | 0.9352(0.9213, 0.9492) | 0.7110(0.6535, 0.7685) | 0.4524(0.3333, 0.5714) | 0.9631(0.9508, 0.9754) | 0.4105(0.2941, 0.5268) | 0.4204(0.3145, 0.5263) |
| SPU-10 | 0.9688(0.9570, 0.9805) | 0.7417(0.6559, 0.8276) | 0.4986(0.3305, 0.6667) | 0.9918(0.9876, 0.9960) | 0.7670(0.6250, 0.9091) | 0.5957(0.4545, 0.7368) |
| SR-11 | 0.9495(0.9375, 0.9616) | 0.7573(0.6919, 0.8228) | 0.5417(0.4167, 0.6667) | 0.9734(0.9635, 0.9833) | 0.5256(0.3846, 0.6667) | 0.5232(0.4204, 0.6261) |
| SR-12 | 0.9395(0.9258, 0.9531) | 0.7808(0.7162, 0.8454) | 0.6167(0.5000, 0.7333) | 0.9569(0.9431, 0.9707) | 0.4254(0.3000, 0.5509) | 0.4902(0.3840, 0.5964) |

**Table S7 The performance of the VCUG-DAM for diagnosis L- VUR grading on external validation dataset 1**

|  | **Accuracy**  **(95% CI)** | **AUROC**  **(95% CI)** | **Sensitivity**  **(95% CI)** | **Specificity**  **(95% CI)** | **Precision**  **(95% CI)** | **F1**  **(95% CI)** |
| --- | --- | --- | --- | --- | --- | --- |
| DL | 0.9098(0.8900, 0.9297) | 0.8438(0.7903, 0.8974) | 0.7259(0.6565, 0.7953) | 0.9673(0.9603, 0.9742) | 0.8802(0.8461, 0.9143) | 0.7681(0.7051, 0.8310) |
| **Clinicians without AI model** | | | | | | |
| JPU-1 | 0.8418(0.8203, 0.8633) | 0.6200(0.5712, 0.6687) | 0.5517(0.4905, 0.6129) | 0.9638(0.9583, 0.9693) | 0.5369(0.4647, 0.6092) | 0.5266(0.4715, 0.5818) |
| JPU-2 | 0.8203(0.8002, 0.8405) | 0.6376(0.5839, 0.6914) | 0.5798(0.5183, 0.6413) | 0.9620(0.9564, 0.9676) | 0.5318(0.4833, 0.5803) | 0.5276(0.4856, 0.5696) |
| JR-3 | 0.8571(0.8353, 0.8789) | 0.6707(0.6182, 0.7232) | 0.6001(0.5390, 0.6613) | 0.9663(0.9604, 0.9723) | 0.6010(0.5285, 0.6736) | 0.5766(0.5187, 0.6345) |
| JR-4 | 0.8438(0.8236, 0.8639) | 0.6775(0.6274, 0.7277) | 0.6198(0.5626, 0.6769) | 0.9649(0.9592, 0.9706) | 0.5918(0.5268, 0.6568) | 0.5715(0.5274, 0.6156) |
| APU-5 | 0.8884(0.8705, 0.9062) | 0.8314(0.7933, 0.8695) | 0.7889(0.7380, 0.8398) | 0.9743(0.9696, 0.9789) | 0.7561(0.7162, 0.7960) | 0.7388(0.6973, 0.7802) |
| APU-6 | 0.9355(0.9180, 0.9531) | 0.8177(0.7637, 0.8718) | 0.7821(0.7242, 0.8400) | 0.9862(0.9825, 0.9900) | 0.8648(0.8239, 0.9056) | 0.8046(0.7535, 0.8557) |
| AR-7 | 0.8636(0.8438, 0.8834) | 0.7941(0.7436, 0.8446) | 0.7689(0.7133, 0.8245) | 0.9739(0.9696, 0.9782) | 0.6386(0.5817, 0.6954) | 0.6546(0.5986, 0.7106) |
| AR-8 | 0.9040(0.8900, 0.9180) | 0.8811(0.8435, 0.9188) | 0.8684(0.8265, 0.9103) | 0.9830(0.9802, 0.9858) | 0.7077(0.6599, 0.7554) | 0.7558(0.7111, 0.8005) |
| SPU-9 | 0.8890(0.8711, 0.9069) | 0.7648(0.6960, 0.8335) | 0.7087(0.6358, 0.7817) | 0.9756(0.9706, 0.9807) | 0.6595(0.5916, 0.7275) | 0.6670(0.6013, 0.7326) |
| SPU-10 | 0.8770(0.8555, 0.8984) | 0.7431(0.6939, 0.7923) | 0.7143(0.6597, 0.7688) | 0.9741(0.9692, 0.9790) | 0.6581(0.5881, 0.7281) | 0.6449(0.5848, 0.7050) |
| SR-11 | 0.9238(0.9102, 0.9375) | 0.8657(0.8305, 0.9009) | 0.8278(0.7793, 0.8763) | 0.9823(0.9778, 0.9867) | 0.7847(0.7313, 0.8380) | 0.7962(0.7528, 0.8396) |
| SR-12 | 0.9199(0.9023, 0.9375) | 0.8859(0.8620, 0.9097) | 0.8567(0.8176, 0.8958) | 0.9845(0.9806, 0.9884) | 0.7310(0.6761, 0.7858) | 0.7690(0.7220, 0.8159) |
| **Clinicians with AI model** | | | | | | |
| JPU-1 | 0.8962(0.8783, 0.9141) | 0.7428(0.6921, 0.7934) | 0.6892(0.6291, 0.7493) | 0.9736(0.9685, 0.9786) | 0.7190(0.6526, 0.7853) | 0.6775(0.6135, 0.7415) |
| JPU-2 | 0.9141(0.8978, 0.9303) | 0.7994(0.7540, 0.8449) | 0.7591(0.7041, 0.8140) | 0.9788(0.9744, 0.9833) | 0.7432(0.6962, 0.7901) | 0.7363(0.6869, 0.7857) |
| JR-3 | 0.9336(0.9180, 0.9492) | 0.8418(0.8076, 0.8760) | 0.7913(0.7456, 0.8370) | 0.9805(0.9758, 0.9852) | 0.8603(0.8155, 0.9050) | 0.7979(0.7502, 0.8457) |
| JR-4 | 0.9241(0.9102, 0.9381) | 0.8520(0.8008, 0.9032) | 0.8114(0.7505, 0.8722) | 0.9793(0.9742, 0.9844) | 0.7793(0.7189, 0.8396) | 0.7793(0.7282, 0.8304) |
| APU-5 | 0.9512(0.9375, 0.9648) | 0.9113(0.8739, 0.9487) | 0.8752(0.8253, 0.9250) | 0.9849(0.9805, 0.9893) | 0.8630(0.8270, 0.8990) | 0.8562(0.8110, 0.9014) |
| APU-6 | 0.9746(0.9648, 0.9844) | 0.9570(0.9289, 0.9850) | 0.9491(0.9186, 0.9795) | 0.9953(0.9934, 0.9971) | 0.9447(0.9126, 0.9768) | 0.9442(0.9171, 0.9713) |
| AR-7 | 0.9590(0.9453, 0.9727) | 0.9369(0.9018, 0.9720) | 0.9278(0.8885, 0.9671) | 0.9914(0.9887, 0.9942) | 0.8316(0.7757, 0.8876) | 0.8571(0.7995, 0.9146) |
| AR-8 | 0.9727(0.9648, 0.9805) | 0.9434(0.9086, 0.9783) | 0.9366(0.8968, 0.9763) | 0.9949(0.9932, 0.9965) | 0.9194(0.8825, 0.9563) | 0.9158(0.8855, 0.9461) |
| SPU-9 | 0.9707(0.9609, 0.9805) | 0.9514(0.9297, 0.9730) | 0.9270(0.8968, 0.9573) | 0.9918(0.9886, 0.9950) | 0.9381(0.9093, 0.9668) | 0.9279(0.8997, 0.9562) |
| SPU-10 | 0.9746(0.9648, 0.9844) | 0.9302(0.8942, 0.9662) | 0.9190(0.8789, 0.9590) | 0.9916(0.9884, 0.9947) | 0.9257(0.8903, 0.9610) | 0.9110(0.8715, 0.9506) |
| SR-11 | 0.9785(0.9688, 0.9883) | 0.9417(0.9055, 0.9780) | 0.9222(0.8775, 0.9670) | 0.9925(0.9892, 0.9958) | 0.9665(0.9423, 0.9908) | 0.9387(0.9065, 0.9710) |
| SR-12 | 0.9805(0.9727, 0.9883) | 0.9864(0.9757, 0.9971) | 0.9822(0.9700, 0.9944) | 0.9966(0.9953, 0.9980) | 0.9245(0.8907, 0.9583) | 0.9456(0.9179, 0.9733) |

**Table S8 The performance of the VCUG-DAM for diagnosis R-VUR grading on external validation dataset 1**

|  | **Accuracy**  **(95% CI)** | **AUROC**  **(95% CI)** | **Sensitivity**  **(95% CI)** | **Specificity**  **(95% CI)** | **Precision**  **(95% CI)** | **F1**  **(95% CI)** |
| --- | --- | --- | --- | --- | --- | --- |
| **DL** | 0.8965(0.8750, 0.9180) | 0.9104(0.8825, 0.9383) | 0.7539(0.6878, 0.8200) | 0.9699(0.9624, 0.9775) | 0.8049(0.7411, 0.8686) | 0.7520(0.6836, 0.8203) |
| **Clinicians without AI model** | | | | | | |
| JPU-1 | 0.8675(0.8477, 0.8873) | 0.6998(0.6496, 0.7500) | 0.6169(0.5498, 0.6839) | 0.9684(0.9624, 0.9743) | 0.6118(0.5413, 0.6823) | 0.5873(0.5231, 0.6515) |
| JPU-2 | 0.8825(0.8627, 0.9023) | 0.8000(0.7579, 0.8422) | 0.7340(0.6824, 0.7855) | 0.9714(0.9662, 0.9766) | 0.6465(0.5854, 0.7076) | 0.6700(0.6145, 0.7254) |
| JR-3 | 0.8688(0.8509, 0.8867) | 0.7109(0.6568, 0.7651) | 0.6310(0.5734, 0.6886) | 0.9705(0.9659, 0.9751) | 0.5914(0.5092, 0.6737) | 0.5882(0.5180, 0.6584) |
| JR-4 | 0.8558(0.8398, 0.8717) | 0.7501(0.7018, 0.7984) | 0.6757(0.6242, 0.7273) | 0.9670(0.9636, 0.9705) | 0.5658(0.5155, 0.6161) | 0.6022(0.5547, 0.6498) |
| APU-5 | 0.8988(0.8828, 0.9147) | 0.8227(0.7761, 0.8693) | 0.7725(0.7141, 0.8309) | 0.9753(0.9697, 0.9808) | 0.6744(0.6221, 0.7266) | 0.7063(0.6552, 0.7574) |
| APU-6 | 0.8942(0.8783, 0.9102) | 0.7978(0.7475, 0.8480) | 0.7367(0.6733, 0.8001) | 0.9749(0.9699, 0.9799) | 0.6879(0.6277, 0.7481) | 0.6867(0.6247, 0.7487) |
| AR-7 | 0.8532(0.8314, 0.8750) | 0.8405(0.7961, 0.8848) | 0.8100(0.7619, 0.8582) | 0.9722(0.9675, 0.9768) | 0.6139(0.5634, 0.6645) | 0.6599(0.6030, 0.7168) |
| AR-8 | 0.8789(0.8594, 0.8984) | 0.8608(0.8208, 0.9008) | 0.8107(0.7532, 0.8681) | 0.9763(0.9724, 0.9801) | 0.6689(0.6206, 0.7172) | 0.7090(0.6641, 0.7538) |
| SPU-9 | 0.8750(0.8555, 0.8945) | 0.8270(0.7794, 0.8745) | 0.7820(0.7189, 0.8451) | 0.9758(0.9715, 0.9802) | 0.6715(0.6184, 0.7246) | 0.6960(0.6444, 0.7477) |
| SPU-10 | 0.8789(0.8627, 0.8952) | 0.7761(0.7293, 0.8228) | 0.7004(0.6464, 0.7544) | 0.9748(0.9708, 0.9788) | 0.6035(0.5497, 0.6573) | 0.6354(0.5773, 0.6935) |
| SR-11 | 0.9316(0.9141, 0.9492) | 0.9216(0.8853, 0.9579) | 0.8929(0.8381, 0.9478) | 0.9863(0.9815, 0.9911) | 0.8088(0.7652, 0.8525) | 0.8318(0.7860, 0.8776) |
| SR-12 | 0.9124(0.8984, 0.9264) | 0.8689(0.8387, 0.8992) | 0.8337(0.7925, 0.8749) | 0.9825(0.9784, 0.9866) | 0.7403(0.6765, 0.8041) | 0.7706(0.7236, 0.8176) |
| **Clinicians with AI model** | | | | | | |
| JPU-1 | 0.9124(0.8945, 0.9303) | 0.7869(0.7404, 0.8333) | 0.7361(0.6785, 0.7938) | 0.9800(0.9752, 0.9847) | 0.7546(0.6933, 0.8158) | 0.7047(0.6423, 0.7672) |
| JPU-2 | 0.9199(0.9023, 0.9375) | 0.8469(0.8101, 0.8837) | 0.7887(0.7362, 0.8413) | 0.9794(0.9743, 0.9844) | 0.7587(0.6850, 0.8324) | 0.7539(0.7006, 0.8072) |
| JR-3 | 0.9222(0.9062, 0.9381) | 0.8340(0.7936, 0.8745) | 0.7828(0.7276, 0.8381) | 0.9820(0.9778, 0.9863) | 0.7538(0.6917, 0.8158) | 0.7269(0.6637, 0.7900) |
| JR-4 | 0.9043(0.8867, 0.9219) | 0.8566(0.8133, 0.8999) | 0.8054(0.7517, 0.8590) | 0.9775(0.9728, 0.9821) | 0.7057(0.6530, 0.7584) | 0.7352(0.6864, 0.7839) |
| APU-5 | 0.9238(0.9062, 0.9414) | 0.8820(0.8409, 0.9230) | 0.8521(0.8052, 0.8989) | 0.9828(0.9784, 0.9872) | 0.7525(0.7019, 0.8031) | 0.7863(0.7442, 0.8284) |
| APU-6 | 0.9336(0.9180, 0.9492) | 0.8902(0.8459, 0.9346) | 0.8633(0.8084, 0.9182) | 0.9844(0.9800, 0.9889) | 0.7989(0.7411, 0.8566) | 0.8197(0.7749, 0.8646) |
| AR-7 | 0.9180(0.8984, 0.9375) | 0.9377(0.9085, 0.9670) | 0.9248(0.8912, 0.9585) | 0.9856(0.9822, 0.9891) | 0.7501(0.6958, 0.8045) | 0.7989(0.7485, 0.8493) |
| AR-8 | 0.9395(0.9258, 0.9531) | 0.9198(0.8842, 0.9554) | 0.9057(0.8644, 0.9471) | 0.9893(0.9867, 0.9919) | 0.7777(0.7205, 0.8349) | 0.8164(0.7607, 0.8720) |
| SPU-9 | 0.9297(0.9141, 0.9453) | 0.9203(0.8766, 0.9641) | 0.9010(0.8500, 0.9520) | 0.9855(0.9812, 0.9898) | 0.8388(0.7861, 0.8914) | 0.8523(0.8032, 0.9014) |
| SPU-10 | 0.9434(0.9297, 0.9570) | 0.9136(0.8827, 0.9445) | 0.8785(0.8302, 0.9269) | 0.9879(0.9848, 0.9909) | 0.8242(0.7738, 0.8747) | 0.8346(0.7908, 0.8784) |
| SR-11 | 0.9652(0.9531, 0.9772) | 0.9664(0.9404, 0.9925) | 0.9594(0.9299, 0.9889) | 0.9938(0.9917, 0.9960) | 0.9002(0.8591, 0.9413) | 0.9189(0.8830, 0.9548) |
| SR-12 | 0.9509(0.9369, 0.9648) | 0.9347(0.9037, 0.9658) | 0.9269(0.8921, 0.9618) | 0.9913(0.9888, 0.9937) | 0.8270(0.7687, 0.8853) | 0.8634(0.8185, 0.9082) |

**Table S9 The performance of the VCUG-DAM for Left VUR grading on the external validation dataset2**

|  | **Accuracy**  **(95% CI)** | **Precision**  **(95% CI)** | **Sensitivity**  **(95% CI)** | **F1**  **(95% CI)** | **AUROC**  **(95% CI)** | **Specificity**  **(95% CI)** |
| --- | --- | --- | --- | --- | --- | --- |
| **DL** | 0.8769(0.8433, 0.9104) | 0.8812(0.8327,0.9296) | 0.8291(0.7816, 0.8765) | 0.8435(0.7943, 0.8926) | 0.8995(0.8716, 0.9274) | 0.9691(0.9614, 0.9767) |
| **Clinicians without AI model** |  |  |  |  |  |  |
| JPU-1 | 0.7537(0.7164, 0.7910) | 0.6335(0.5706, .6964) | 0.7139(0.6398, 0.7879) | 0.6398(0.5765, 0.7032) | 0.8325(0.7936, 0.8714) | 0.9516(0.9439, 0.9593) |
| JPU-2 | 0.8120(0.7612, 0.8629) | 0.6877(0.6246, .7508) | 0.7069(0.6342, 0.7796) | 0.6743(0.6151, 0.7336) | 0.8293(0.7911, 0.8676) | 0.9529(0.9451, 0.9608) |
| JR-3 | 0.7845(0.7313, 0.8377) | 0.6884(0.6250, .7518) | 0.7148(0.6225, 0.8071) | 0.6610(0.5770, 0.7450) | 0.8350(0.7848, 0.8852) | 0.9582(0.9482, 0.9683) |
| JR-4 | 0.7761(0.7313, 0.8209) | 0.6591(0.6073,0.7108) | 0.7518(0.6680, 0.8356) | 0.6712(0.6140, 0.7283) | 0.8541(0.8093, 0.8989) | 0.9553(0.9467, 0.9638) |
| APU-5 | 0.8064(0.7687, 0.8442) | 0.6815(0.6300, .7330) | 0.7901(0.7084, 0.8717) | 0.7000(0.6392, 0.7609) | 0.8761(0.8324, 0.9198) | 0.9619(0.9543, 0.9695) |
| APU-6 | 0.8111(0.7789, 0.8433) | 0.6909(0.6476, .7342) | 0.7707(0.6809, 0.8605) | 0.7011(0.6476, 0.7546) | 0.8683(0.8208, 0.9158) | 0.9637(0.9569, 0.9705) |
| AR-7 | 0.7729(0.7313, 0.8144) | 0.6469(0.6014, .6924) | 0.6957(0.6176, 0.7739) | 0.6408(0.5932, 0.6883) | 0.8241(0.7829, 0.8653) | 0.9559(0.9482, 0.9636) |
| AR-8 | 0.7873(0.7509, 0.8237) | 0.6460(0.5888,0.7032) | 0.7135(0.6442, 0.7827) | 0.6516(0.5979, 0.7053) | 0.8344(0.7989, 0.8698) | 0.9582(0.9513, 0.9651) |
| SPU-9 | 0.7924(0.7537, 0.8312) | 0.6862(0.6377, .7347) | 0.7717(0.7045, 0.8388) | 0.6977(0.6458, 0.7495) | 0.8658(0.8303, 0.9013) | 0.9592(0.9517, 0.9666) |
| SPU-10 | 0.8134(0.7687, 0.8582) | 0.7095(0.6483, .7707) | 0.8078(0.7290, 0.8867) | 0.7197(0.6559, 0.7834) | 0.8851(0.8427, 0.9275) | 0.9638(0.9549, 0.9727) |
| SR-11 | 0.8396(0.8060, 0.8731) | 0.7331(0.6893, 0.7770) | 0.8140(0.7451, 0.8828) | 0.7412(0.6928, 0.7896) | 0.8908(0.8543, 0.9274) | 0.9675(0.9603, 0.9747) |
| SR-12 | 0.8298(0.8013, 0.8582) | 0.7207(0.6733, 0.7681) | 0.8050(0.7288, 0.8811) | 0.7384(0.6857, 0.7911) | 0.8864(0.8458, 0.9270) | 0.9668(0.9612, 0.9725) |
| **Clinicians with AI model** | | | | | | |
| JPU-1 | 0.8120(0.7612, 0.8629) | 0.6773(0.6086, 0.7459) | 0.7660(0.6791, 0.8528) | 0.6943(0.6222, 0.7664) | 0.8648(0.8187, 0.9110) | 0.9638(0.9544, 0.9731) |
| JPU-2 | 0.8881(0.8582, 0.9179) | 0.8373(0.7947, 0.8798) | 0.8796(0.8413, 0.9179) | 0.8418(0.8004, 0.8831) | 0.9269(0.9048, 0.9491) | 0.9763(0.9699, 0.9826) |
| JR-3 | 0.8717(0.8358, 0.9076) | 0.7547(0.6897, 0.8196) | 0.7825(0.6939, 0.8712) | 0.7461(0.6728, 0.8193) | 0.8795(0.8331, 0.9260) | 0.9739(0.9670, 0.9809) |
| JR-4 | 0.9104(0.8806, 0.9403) | 0.8085(0.7479, 0.8690) | 0.9245(0.8881, 0.9609) | 0.8406(0.7841, 0.8972) | 0.9544(0.9356, 0.9731) | 0.9831(0.9778, 0.9883) |
| APU-5 | 0.9328(0.9104, 0.9552) | 0.8462(0.7902, 0.9022) | 0.9399(0.9095, 0.9704) | 0.8748(0.8314, 0.9181) | 0.9622(0.9445, 0.9798) | 0.9867(0.9822, 0.9912) |
| APU-6 | 0.9328(0.9104, 0.9552) | 0.8563(0.7972, 0.9154) | 0.9221(0.8881, 0.9561) | 0.8670(0.8102, 0.9238) | 0.9544(0.9366, 0.9723) | 0.9870(0.9826, 0.9914) |
| AR-7 | 0.9478(0.9254, 0.9701) | 0.8964(0.8496, 0.9432) | 0.9311(0.8949, 0.9673) | 0.8963(0.8501, 0.9425) | 0.9598(0.9393, 0.9803) | 0.9890(0.9846, 0.9935) |
| AR-8 | 0.9338(0.9104, 0.9571) | 0.8490(0.7832, 0.9149) | 0.9299(0.8961, 0.9636) | 0.8735(0.8225, 0.9245) | 0.9581(0.9389, 0.9773) | 0.9866(0.9819, 0.9913) |
| SPU-9 | 0.9067(0.8806, 0.9328) | 0.7999(0.7510, 0.8488) | 0.8783(0.8137, 0.9430) | 0.8194(0.7713, 0.8675) | 0.9293(0.8965, 0.9622) | .9806(0.9750, 0.9862) |
| SPU-10 | 0.8918(0.8582, 0.9254) | 0.7894(0.7278, 0.8511) | 0.8910(0.8470, 0.9350) | 0.8172(0.7590, 0.8753) | 0.9348(0.9108, 0.9587) | 0.9787(0.9726, 0.9848) |
| SR-11 | 0.9291(0.9030, 0.9552) | 0.8606(0.7993, 0.9218) | 0.9146(0.8797, 0.9495) | 0.8693(0.8188,0.9199) | 0.9496(0.9301, 0.9691) | 0.9851(0.9800, 0.9902) |
| SR-12 | 0.9478(0.9254, 0.9701) | 0.8823(0.8173,0.9473) | 0.9389(0.9100, 0.9679) | 0.8920(0.8444, 0.9397) | 0.9640(0.9473, 0.9807) | 0.9894(0.9853, 0.9935) |

**Table S10 The performance of the VCUG-DAM for Right VUR grading on the external validation dataset2**

|  | **Accuracy**  **(95% CI)** | **Precision**  **(95% CI)** | **Sensitivity**  **(95% CI)** | **F1**  **(95% CI)** | **AUROC**  **(95% CI)** | **Specificity**  **(95% CI)** |
| --- | --- | --- | --- | --- | --- | --- |
| **DL** | 0.8955(0.8657, 0.9254) | 0.8644(0.8192, 0.9095) | 0.8231(0.7629, 0.8833) | 0.8316(0.7775, 0.8857) | 0.8987(0.8654, 0.9320) | 0.9747(0.9677, 0.9817) |
| **Clinicians without AI model** | | | | | | |
| JPU-1 | 0.7164(0.6716,0.7612) | 0.5136(0.4730, 0.5542) | 0.5597(0.5045, 0.6148) | 0.5048(0.4572, 0.5524) | 0.7520(0.7205, 0.7834) | 0.9420(0.9336, 0.9503) |
| JPU-2 | 0.7052(0.6567,0.7537) | 0.4956(0.4429, 0.5484) | 0.5710(0.4999, 0.6420) | 0.5070(0.4494, 0.5647) | 0.7547(0.7143, 0.7952) | 0.9389(0.9282, 0.9496) |
| JR-3 | 0.7500(0.7015,0.7985) | 0.5525(0.4982, 0.6068) | 0.5996(0.5361, 0.6630) | 0.5589(0.5013, 0.6165) | 0.7753(0.7412, 0.8095) | 0.9482(0.9387, 0.9578) |
| JR-4 | 0.7127(0.6791,0.7463) | 0.5292(0.4800, 0.5785) | 0.5863(0.5300, 0.6426) | 0.5293(0.4773, 0.5812) | 0.7634(0.7340, 0.7929) | 0.9405(0.9336, 0.9474) |
| APU-5 | 0.7463(0.7071, 0.7854) | 0.5734(0.5150, 0.6319) | 0.6009(0.5479, 0.6539) | 0.5520(0.5005, 0.6035) | 0.7746(0.7452, 0.8040) | 0.9474(0.9389, 0.9559) |
| APU-6 | 0.7687(0.7313, 0.8060) | 0.5823(0.5298, 0.6347) | 0.6236(0.5645, 0.6827) | 0.5840(0.5347, 0.6333) | 0.7863(0.7539, 0.8187) | 0.9529(0.9449, 0.9609) |
| AR-7 | 0.7425(0.7015, 0.7836) | 0.5306(0.4897, 0.5715) | 0.5799(0.5285, 0.6314) | 0.5360(0.4879, 0.5841) | 0.7634(0.7361, 0.7907) | 0.9457(0.9378, 0.9537) |
| AR-8 | 0.7276(0.6866, 0.7687) | 0.5095(0.4568, 0.5623) | 0.5657(0.5064, 0.6251) | 0.5200(0.4611, 0.5790) | 0.7530(0.7174, 0.7886) | 0.9435(0.9345, 0.9525) |
| SPU-9 | 0.7090(0.6642, 0.7537) | 0.5244(0.4631, 0.5856) | 0.5303(0.4690, 0.5916) | 0.5105(0.4600, 0.5609) | 0.7352(0.7017, 0.7687) | 0.9387(0.9303, 0.9471) |
| SPU-10 | 0.7677(0.7295, 0.8060) | 0.5821(0.5288, 0.6355) | 0.6289(0.5736, 0.6841) | 0.5897(0.5405, 0.6390) | 0.7885(0.7577, 0.8193) | 0.9513(0.9432, 0.9593) |
| SR-11 | 0.7687(0.7313, 0.8060) | 0.5780(0.5241, 0.6318) | 0.6223(0.5644, 0.6802) | 0.5750(0.5239, 0.6262) | 0.7874(0.7559, 0.8189) | 0.9528(0.9444, 0.9612) |
| SR-12 | 0.7761(0.7313, 0.8209) | 0.6113(0.5534, 0.6693) | 0.6430(0.5956, 0.6905) | 0.6036(0.5489, 0.6583) | 0.8002(0.7733, 0.8271) | 0.9546(0.9467, 0.9625) |
| **Clinicians with AI model** | | | | | | |
| JPU-1 | 0.7980(0.7537, 0.8424) | 0.5739(0.5252, 0.6226) | 0.6187(0.5648, 0.6726) | 0.5832(0.5330, 0.6334) | 0.7897(0.7609, 0.8185) | 0.9594(0.9516, 0.9672) |
| JPU-2 | 0.7887(0.7463, 0.8312) | 0.5672(0.5247, 0.6096) | 0.6628(0.6154, 0.7101) | 0.5844(0.5360, 0.6328) | 0.8103(0.7822, 0.8384) | 0.9574(0.9485, 0.9662) |
| JR-3 | 0.8731(0.8433, 0.9030) | 0.6724(0.6215, 0.7233) | 0.6978(0.6536, 0.7420) | 0.6730(0.6229, 0.7230) | 0.8352(0.8125, 0.8578) | 0.9722(0.9653, 0.9790) |
| JR-4 | 0.8284(0.7910, 0.8657) | 0.6173(0.5821, 0.6524) | 0.6894(0.6382, 0.7406) | 0.6284(0.5904, 0.6664) | 0.8281(0.7994, 0.8567) | 0.9661(0.9590, 0.9733) |
| APU-5 | 0.8582(0.8284, 0.8881) | 0.6657(0.6145, 0.7169) | 0.7240(0.6738, 0.7742) | 0.6722(0.6300, 0.7143) | 0.8488(0.8238, 0.8739) | 0.9715(0.9648, 0.9783) |
| APU-6 | 0.8396(0.8013, 0.8778) | 0.6073(0.5542, 0.6605) | 0.6683(0.6145, 0.7220) | 0.6202(0.5649, 0.6755) | 0.8173(0.7874, 0.8472) | 0.9658(0.9588, 0.9728) |
| AR-7 | 0.8475(0.8069, 0.8881) | 0.6500(0.6064, 0.6936) | 0.7135(0.6642, 0.7628) | 0.6697(0.6268, 0.7125) | 0.8411(0.8132, 0.8690) | 0.9679(0.9595, 0.9764) |
| AR-8 | 0.8727(0.8358, 0.9095) | 0.6504(0.5975, 0.7034) | 0.7051(0.6579, 0.7523) | 0.6654(0.6109, 0.7199) | 0.8396(0.8124, 0.8668) | 0.9736(0.9668, 0.9804) |
| SPU-9 | 0.8349(0.8041, 0.8657) | 0.6205(0.5535, 0.6874) | 0.6364(0.5718, 0.7010) | 0.6119(0.5464, 0.6774) | 0.8002(0.7659, 0.8346) | 0.9661(0.9597, 0.9725) |
| SPU-10 | 0.8209(0.7836, 0.8582) | 0.6338(0.5742, 0.6934) | 0.6429(0.5703, 0.7155) | 0.6261(0.5659, 0.6864) | 0.8030(0.7623, 0.8438) | 0.9625(0.9538, 0.9713) |
| SR-11 | 0.8470(0.8134, 0.8806) | 0.6375(0.5844, 0.6907) | 0.6787(0.6113, 0.7461) | 0.6456(0.5961, 0.6952) | 0.8234(0.7863, 0.8605) | 0.9682(0.9601, 0.9764) |
| SR-12 | 0.8731(0.8507, 0.8955) | 0.6587(0.6113, 0.7060) | 0.7390(0.6913, 0.7867) | 0.6805(0.6291, 0.7319) | 0.8575(0.8329, 0.8822) | 0.9742(0.9681, 0.9802) |

**Table S11 Basic information of 12 clinicians**

| **No** | **Name** | **The title of a professional post** | **Hospital** |
| --- | --- | --- | --- |
| **1** | Tao Zhang | Resident pediatric urologists-1 | Department of Urology, Huai'an Ruiji Hospital |
| **2** | Siyi Zhou | Resident pediatric urologists-2 | Department of Urology, Children′s Hospital of Fudan University |
| **3** | Yubo Sun | Attending pediatric urologists-1 | Department of Urology, Children′s Hospital of Fudan University |
| **4** | Hongwang Xue | Attending pediatric urologists-2 | Department of Urology, Huai'an First People's Hospital |
| **5** | Ying Liu | Senior pediatric urologists-1 | Department of Urology, Children′s Hospital of Fudan University |
| **6** | Weijing Ye | Senior pediatric urologists-2 | Department of Urology, Renji Hospital, School of Medicine, Shanghai Jiao Tong University |
| **7** | Xiang Ren | Resident pediatric radiologists-1 | Department of Radiology, Shanghai Children's Hospital, School of medicine, Shanghai Jiao Tong University |
| **8** | Xin Zhao | Resident pediatric radiologists-2 | Department of Radiology, Children′s Hospital of Fudan University |
| **9** | Qiong Yao | Attending pediatric radiologists-1 | Department of Radiology, Children′s Hospital of Fudan University |
| **10** | Jinhua Fu | Attending pediatric radiologists-2 | Department of Radiology, Shanghai Children's Hospital, School of medicine, Shanghai Jiao Tong University |
| **11** | Min Ji | Senior pediatric radiologists-1 | Department of Radiology, Children′s Hospital of Fudan University |
| **12** | Haifeng Lu | Senior pediatric radiologists-2 | Department of Radiology, Huai'an Second People's Hospital |

**TRIPOD check list.**

| **Section and Topic** | **Item #** | **Development or validation?** | **Checklist item** | **Page** |
| --- | --- | --- | --- | --- |
| **Title or abstract** | | |  |  |
| **Title** | 1 | D;V | Identify the study as developing and/or validating a multivariable prediction model, the target population, and the outcome to be predicted. | Page 1 |
| **abstract** | 2 | D;V | Provide a summary of objectives, study design, setting, participants, sample size, predictors, outcome, statistical analysis, results, and conclusions. | Page 2 |
| **INTRODUCTION** | | |  |  |
| **Background and objectives** | 3a | D;V | Explain the medical context (including whether diagnostic or prognostic) and rationale for developing or validating  the multivariable prediction model, including references to existing models. | Page 3 |
|  | 3b | D;V | Specify the objectives, including whether the study describes the development or validation of the model, or both. | Page 3 |
| **METHODS** | | |  |  |
| **Source of data** | 4a | D;V | Describe the study design or source of data (e.g., randomized trial, cohort, or registry data), separately for the development and validation data sets, if applicable. | Page 16,  Supplemental Figure S1 |
|  | 4b | D;V | Specify the key study dates, including start of accrual; end of accrual; and, if applicable, end of follow-up | Supplemental File S1 and Figure S2 |
| **Participants** | 5a | D;V | Specify key elements of the study setting (e.g., primary care, secondary care, general population) including number and location of centres. | Supplemental File S1 and Figure S2 |
|  | 5b | D;V | Describe eligibility criteria for participants. | Supplemental File 1 |
|  | 5c | D;V | Give details of treatments received, if relevant. | - |
| **Outcome** | 6a | D;V | Clearly define the outcome that is predicted by the prediction Clearly define the outcome that is predicted by the prediction | - |
|  | 6b | D;V | Report any actions to blind assessment of the outcome to be predicted. | - |
| **Predictors** | 7a | D;V | Clearly define all predictors used in developing the multivariable prediction model, including how and when they were measured. | - |
|  | 7b | D;V | Report any actions to blind assessment of predictors for the outcome and other predictors. | - |
| **Sample size** | 8 | D;V | Explain how the study size was arrived at. | Supplemental File 3 |
| **Missing data** | 9 | D;V | Describe how missing data were handled (e.g., complete-case analysis, single imputation, multiple imputation) with details of any imputation method. | Supplemental File 1 |
| **Statistical analysis methods** | 10a | D | Describe how predictors were handled in the analyses. | - |
|  | 10b | D | Specify type of model, all model-building procedures (including any predictor selection), and method for internal validation. | Supplemental File 3 |
|  | 10c | V | For validation, describe how the predictions were calculated. | - |
|  | 10d | D;V | Specify all measures used to assess model performance and, if relevant, to compare multiple models. | - |
|  | 10e | V | Describe any model updating (e.g., recalibration) arising from the validation, if done. | - |
| **Risk groups** | 11 | D;V | Provide details on how risk groups were created, if done | **-** |
| **Development vs. validation** | 12 | V | For validation, identify any differences from the development data in setting, eligibility criteria, outcome, and predictors. | - |
| **RESULTS** | | |  |  |
| **Participants** | 13a | D;V | Describe the flow of participants through the study, including the number of participants with and without the outcome and, if applicable, a summary of the follow-up time. A diagram may be helpful. | Supplementary figure S1 |
|  | 13b | D;V | Describe the characteristics of the participants (basic demographics, clinical features, available predictors), including the number of participants with missing data for predictors and outcome. | Tabel 1 |
|  | 13c | V | For validation, show a comparison with the development data of the distribution of important variables (demographics, predictors and outcome). | Tabel 1 |
| **Model development** | 14a | D | Specify the number of participants and outcome events in each analysis. | - |
|  | 14b | D | If done, report the unadjusted association between each candidate predictor and outcome. | - |
| **Model specification** | 15a | D | Present the full prediction model to allow predictions for individuals (i.e., all regression coefficients, and model intercept or baseline survival at a given time point). | - |
|  | 15b | D | Explain how to use the prediction model. | Fig.4, Fig.5 |
| **Model performance** | 16 | D;V | Report performance measures (with CIs) for the prediction model. | Fig.2;  Supplementary TablesS3-S10 |
| **Model updating** | 17 | V | If done, report the results from any model updating (i.e., model specification, model performance). | - |
| Discussion | | |  |  |
| **Limitations** | 18 | D;V | Discuss any limitations of the study (such as nonrepresentative sample, few events per predictor, missing data). | Page 15 |
| **Interpretation** | 19a | V | For validation, discuss the results with reference to performance in the development data, and any other validation data. | Page 13 to15 |
|  | 19b | D;V | Give an overall interpretation of the results, considering objectives, limitations, results from similar studies, and other relevant evidence. | Page 13 to15 |
| **Implications** | 20 | D;V | Discuss the potential clinical use of the model and implications for future research. | Page 15 |
| **Other information** |  |  |  |  |
| **Supplementary information** | 21 | D;V | Provide information about the availability of supplementary resources, such as study protocol, Web calculator, and data sets. | Supplementary Figure S1  Page 17 |
| **Funding** | 22 | D;V | Give the source of funding and the role of the funders for the present study. | Page 19 |
